# Targeting MS4A4A: A novel pathway to improve immunotherapy responses in glioblastoma

**DOI:** 10.1111/cns.14791

**Published:** 2024-07-12

**Authors:** Guangcai Shao, Xiangguo Cui, Yiliang Wang, Shuyan Luo, Chuanyu Li, Yu Jiang, Dasheng Cai, Nu Li, Xiang Li

**Affiliations:** ^1^ Department of Neurosurgery, Shengjing Hospital China Medical University Shenyang China; ^2^ Department of Neurosurgery Anshan Central Hospital Anshan China; ^3^ Department of Otolaryngology Head and Neck Surgery, Shengjing Hospital China Medical University Shenyang China; ^4^ Department of Anesthesiology The First Hospital of China Medical University Shenyang China; ^5^ Department of Neurosurgery The First Hospital of China Medical University Shenyang China; ^6^ Department of Breast Surgery The First Hospital of China Medical University Shenyang China

**Keywords:** ferroptosis pathway, glioblastoma, immune checkpoint blockade, immunotherapy, macrophage polarization, MS4A4A

## Abstract

**Introduction:**

Glioblastoma (GBM) remains a challenging brain tumor to treat, with limited response to PD‐1 immunotherapy due to tumor‐associated macrophages (TAMs), specifically the M2 phenotype. This study explores the potential of MS4A4A (membrane spanning four domains, subfamily A, member 4A) inhibition in driving M2 macrophage polarization toward the M1 phenotype via the ferroptosis pathway to enhance the effectiveness of immunotherapy in GBM.

**Methods:**

Single‐cell RNA sequencing and spatial transcriptomic analyses were employed to characterize M2 macrophages and MS4A4A expression in GBM. In vitro studies utilizing TAM cultures, flow cytometry, and western blot validations were conducted to assess the impact of MS4A4A on the tumor immune microenvironment and M2 macrophage polarization. In vivo models, including subcutaneous and orthotopic transplantation in mice, were utilized to evaluate the effects of MS4A4A knockout and combined immune checkpoint blockade (ICB) therapy on tumor growth and response to PD‐1 immunotherapy.

**Results:**

Distinct subsets of GBM‐associated macrophages were identified, with spatial distribution in tumor tissue elucidated. In vivo experiments demonstrated that inhibiting MS4A4A and combining ICB therapy effectively inhibited tumor growth, reshaped the tumor immune microenvironment by reducing M2 TAM infiltration and enhancing CD8^+^ T‐cell infiltration, ultimately leading to complete tumor eradication.

**Conclusion:**

MS4A4A inhibition shows promise in converting M2 macrophages to M1 phenotype via ferroptosis, decreasing M2‐TAM infiltration, and enhancing GBM response to PD‐1 immunotherapy. These findings offer a novel approach to developing more effective immunotherapeutic strategies for GBM.

## Graphical abstract

This study employs single‐cell transcriptomic analysis to identify subgroups of glioblastoma‐associated macrophages. Spatial transcriptomic analysis reveals the spatial distribution of macrophages within glioblastoma tissue. In vitro experiments demonstrate that inhibition of MS4A4A reduces the immunosuppressive function of M2‐type macrophages. In vivo experiments confirm that inhibition of MS4A4A enhances the response of glioblastoma to PD‐1 immunotherapy. This study provides new theoretical bases and molecular therapeutic targets for the diagnosis and treatment of glioblastoma.

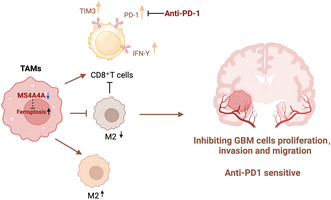

## 1 INTRODUCTION

Glioblastoma (GBM) is a highly invasive and recurrent brain tumor, representing one of the leading causes of tumor‐related deaths in the brain.^1^, ^2^, ^3^ As a novel treatment approach, immunotherapy has demonstrated remarkable effectiveness in certain patients diagnosed with gliomas.^3^, ^4^, ^5^, ^6^ Macrophages expressing various anti‐inflammatory cytokines such as IL‐10, TGF‐β, and inhibitory factors produced by multiple inflammatory stimuli and participating in tissue repair and recycling processes have been identified.^7^, ^8^ However, in the tumor microenvironment (TME), M2 macrophages transform into M2‐type tumor‐associated macrophages (M2‐TAMs), oversecreting various cytokines to promote the growth, migration, and invasion of tumor cells while inhibiting the immune system response.^9^, ^10^ In the microenvironment of GBM, a highly aggressive malignant tumor, there is a significant increase in the number and widespread distribution of M2 macrophages and M2‐TAMs. Recent studies have indicated that EVs released by GBM can enhance the expression of PD‐L1 in TAMs.^11^ PD‐1 immune checkpoint inhibitors are currently one of the most frequently utilized drugs in immunotherapy. However, their therapeutic effectiveness is still limited.^12^, ^13^ M2 macrophages, immune‐suppressive cell types in the tumor immune microenvironment, could hamper the efficacy of PD‐1 immunotherapy.^14^, ^15^, ^16^ This study investigates whether inhibiting MS4A4A (membrane spanning four domains, subfamily A, member 4A) could decrease the infiltration of M2‐TAMs. The objective is to modify the tumor immune microenvironment and improve the responsiveness of GBM to PD‐1 immunotherapy.

MS4A4A is a cell surface protein predominantly expressed in diverse immune system cells, such as macrophages and T cells. It is crucial in numerous biological processes, including cell activation, proliferation, and intercellular communication.^17^, ^18^, ^19^ In highly invasive tumor samples, a significant upregulation of MS4A4A can be observed. Studies have indicated that MS4A4A promotes the necessary proliferation and regenerative capacity of neural stem cells for GBM through the STAT3 pathway.^20^, ^21^ Prior research has demonstrated that MS4A4A regulated the function and polarization of macrophages.^20^ However, there is still a relatively limited amount of research on regulating macrophage function by MS4A4A in gliomas. Therefore, we hypothesize that the suppression of MS4A4A could impact the function of M2 macrophages, resulting in alterations to the immune microenvironment and ultimately enhancing the effectiveness of PD‐1 immunotherapy.

This study utilized single‐cell and spatial transcriptomic sequencing techniques to further explore the effects of inhibiting MS4A4A on M2 macrophages. Single‐cell sequencing allows the detection of transcriptional variations in distinct cell subpopulations, identifying alterations in gene expression, specifically in M2 macrophages.^22^, ^23^ Transcriptome sequencing of spatially resolved samples could uncover interactions between macrophages and other cell types at the tissue level. This approach offers a more comprehensive understanding of the intricacies of the immune microenvironment in gliomas.

This study aims to enhance our understanding of the role and mechanism of MS4A4A in the immune microenvironment of GBM. Inhibition of MS4A4A could reduce M2‐TAM infiltration, regulate gene expression about ferroptosis, and improve the responsiveness of PD‐1 immunotherapy by restructuring the tumor immune microenvironment. This study will offer novel research ideas for developing immunotherapy strategies for glioma, contributing to positive scientific and clinical advancements in enhancing glioma patients' survival rate and quality of life during clinical treatment. By comprehending the functional regulatory mechanisms of M2 macrophages within the immune microenvironment of glioma, we aim to establish a basis for developing novel targeted treatment strategies and combination therapy options. It will ultimately enhance the treatment options available to glioma patients, leading to more effective outcomes.

## 2 MATERIALS AND METHODS

### 2.1 Declaration of ethical principles for animal research

All animal experiments in this study complied with the regulations and guidelines set by our institution's animal experimentation ethics committee and received the necessary approval. Every effort is made in experimental research to minimize pain and distress in animals and reduce the number of animals required for experiments to the greatest extent possible. Animal breeding, handling, and experimental operations adhere to internationally recognized animal welfare standards. We ensure that all animals receive appropriate care and are properly disposed of after completing the experiments.

### 2.2 Download and analysis of single‐cell sequencing data obtained from tumor tissue samples in patients with GBM

The GSE235676 dataset was downloaded from the Gene Expression Omnibus (GEO) database (http://www.ncbi.nlm.nih.gov/geo/). This dataset contains single‐cell RNA sequencing (scRNA‐seq) data of four GBM tumor types. In this study, we selected 12 single‐cell sequencing samples from the dataset. These samples were divided into four groups: three cases of newly diagnosed glioma patients (ND) (GSM7507509, GSM7507514, and GSM7507518), three cases of patients who relapsed after receiving temozolomide (TMZ) and radiotherapy (Rec) (GSM7507508, GSM7507511, and GSM7507513), three cases of patients who did not respond to the combination therapy of pembrolizumab (PD‐1 inhibitor) and anlotinib in the neoadjuvant setting (Non) (GSM7507507, GSM7507516, and GSM7507522), and three cases of patients who responded to the combination therapy of pembrolizumab (PD‐1 inhibitor) and anlotinib in the neoadjuvant setting (Res) (GSM7507523, GSM7507524, and GSM7507525). Analyze the data from 12 glioma samples using the “Seurat” package in the R software. We conducted quality control on the data using 200 < nFeature_RNA < 5000 percent criteria.mt < 5. We then selected highly variable genes based on the top 2000 genes with the highest variance.^24^

### 2.3 UMAP clustering analysis and cell annotation

To decrease the dimensionality of the scRNA‐Seq dataset, we conducted principal component analysis (PCA) on the 2000 most variable genes, chosen based on their maximum variance. The downstream analysis utilized the first 14 principal components (PCs) selected by the Elbowplot function of the Seurat package. Major cell subpopulations were identified using the FindClusters function provided by Seurat, with the resolution set to the default value (resolution = 0.2). Subsequently, the UMAP algorithm was applied to reduce nonlinear dimensionality of scRNA‐seq sequencing data. Finally, we utilized established marker genes specific to cell lineages and annotated the cells using the online resource CellMarker.^25^

### 2.4 Monocle temporal analysis

The experiment aims to use Monocle (version 2.18.0) to analyze single‐cell transcriptome data and infer the developmental trajectory of macrophages. To begin with, the macrophages need to be isolated from the Seurat object. Then, the cell data should be transformed into the SingleCellExperiment format, which is widely used for storing single‐cell RNA‐Seq data. Follow the official Monocle tutorial for trajectory analysis. Convert data in SingleCellExperiment format to a Monocle object using the new CellDataSet function available in Monocle. Utilize the Estimate Size Factors function to estimate the size factors for all cellular components. Next, the Dispersions function should be used to estimate the dispersion of all genes. Choose genes expressed in more than 10 cells with an average expression level higher than 0.1 for further analysis. This study aims to examine differential gene expression among various subtypes of macrophages by employing statistical tests. To generate a pseudotime trajectory, cells were sorted based on differentially expressed genes (DEGs) with *q* values less than 0.01.^26^

### 2.5 Differential gene analysis

The “Limma” package in R software filters DEGs in scRNA‐Seq datasets. DEGs were selected based on the criteria of |logFC| > 0.85 and *p*.adjust < 0.05 between the patients who did not respond (Non) to the combination therapy of pembrolizumab (PD‐1 inhibitor) and anlotinib, and the three patients (Res) who did respond to this therapy.^26^

### 2.6 Downloading and analyzing spatial transcriptome data from tumor tissue samples in patients with GBM

To access the spatial transcriptome (ST) dataset GSE235672, visit the GEO database at https://www.ncbi.nlm.nih.gov/gds. This study selected four GBM patient tumor tissue slices and their corresponding ST‐seq data from GSE235672. The tissue slices are categorized as ND, Rec, Non, and Res samples, denoted by their respective GSM numbers: GSM7507322, GSM7507317, GSM7507323, and GSM7507327. We integrated the mentioned scRNA‐seq sequencing data with the 10x Visium spatial transcriptomics data from the GSE211895 dataset using the anchor‐based integration pipeline in Seurat. It enables the transfer of cellular type annotations from single‐cell RNA sequencing (scRNA‐seq) to spatial transcriptomics. The predicted cell types in Seurat are imported into an R package called SPOTlight to facilitate the annotation and visualization of cell types at specific spatial locations.^27^

### 2.7 Preparation of conditioned medium

After growing L929 cells to 80% confluence, they were washed twice with phosphate‐buffered saline (PBS), and then 10 mL of Dulbecco's modified Eagle's medium (DMEM) (c11995500BT, Gibco, USA) containing 1% FBS was added. The cells were further cultured for 48 h. Harvest the culture medium, centrifuge it, filter out any cell debris, and retrieve the culture supernatant. To obtain L929‐CM, L929 pretreated medium is mixed with regular culture medium at a volume of ratio 1:4.^28^

The preparation of a tumor‐conditioned medium (CM) involved the following steps. First, glioma cells (CT2A/GL261) were cultured until they reached 80% confluency. Next, the cells were washed twice with PBS and then 10 mL of RPMI 1640 medium (c11875, Gibco, USA) containing 1% FBS was added. The cells were then cultured for an additional 48 h. After that, the medium was collected, centrifuged, and filtered to remove any cell debris. To obtain a CM, the pretreated tumor medium was mixed with the regular culture medium at a 1:1 volume ratio.^28^

### 2.8 Extraction of bone marrow‐derived macrophages from mice

Obtain C57BL/6N mice from our Experimental Animal Research Center, which are 6–8 weeks old and weighed 25–30 g. C57BL/6N mice aged 6–8 weeks were euthanized under anesthesia for this experiment. Subsequently, their bodies were soaked in a 75% ethanol solution for disinfection. Subsequently, scissors were used to incise the skin of the mice's hind limbs, followed by the complete excision of the muscle tissue enveloping each leg bone. During this process, it is crucial to take special care to prevent any harm to the integrity of the joints. Subsequently, the leg bones were sterilized separately using 75% ethanol and PBS. The distal end of the leg bone should be removed. The cells in the bone marrow should then be aspirated and discarded using a syringe and DMEM culture (c11995500BT, Gibco, USA). Afterward, red blood cell lysis buffer should be added, and the mixture should be gently shaken for approximately 2 min.

Next, the lysis process of erythrocytes is stopped by adding DMEM at a volume that is 3–5 times the initial volume. Next, the cells should be centrifuged at a force of 300*g* for 10 min to enhance cell separation and facilitate analysis. After centrifugation, the cells were resuspended in DMEM supplemented with 20% L929 CM.

Once the number of cells is calculated, they are distributed into a six‐well culture plate. Change the culture medium every 3 days. Typically, BMDMs could be induced to reach a mature state after 6–7 days of culture. The cells were labeled with F4/80 and CD11b antibodies to identify them using flow cytometry. The proportion of cells positive for both F4/80 and CD11b exceeded 90%, confirming the successful isolation of BMDMs.^28^, ^29^

### 2.9 Cell culture

The mouse glioma cell lines CT2A and GL261 were purchased from Sigma‐Aldrich (SCC194, Sigma‐Aldrich, USA) and DSMZCellDive (ACC 802, DSMZ, DE), respectively. The human monocyte cell line THP‐1 was obtained from ATCC (TIB‐202, ATCC, USA), and the human embryonic kidney cell line HEK‐293T was acquired from ATCC (CRL‐3216, ATCC, USA). The cultured cells mentioned earlier were maintained in RPMI 1640 complete medium (R4130, Sigma, USA) supplemented with 10% FBS (F8318, Sigma, USA), 100 units/mL penicillin, and 100 μg/mL streptomycin (V900929, Sigma, USA). The incubation was conducted at 37°C in a 5% CO_2_ incubator (Heracell™ Vios 160i CR CO_2_ Incubator, 51033770, Thermo Fisher Scientific™, Germany).

The J774A.1 (TIB‐67) and RAW264.7 (TIB‐208) mouse macrophage cell lines were obtained from ATCC, while the L929 mouse fibroblast cell line was acquired from Shanghai Fusheng Industrial Co., Ltd. (X120311, Shanghai Fusheng Industrial Co., Ltd., CH). The cell lines were cultured in DMEM (11965092, Gibco, USA), supplemented with 10% FBS, 10 μg/mL of streptomycin, and 100 U/mL of penicillin.^30^

### 2.10 Silence and overexpression lentivirus vector construction

The mouse MS4A4A cDNA sequence was analyzed to identify potential target sequences for short hairpin RNA (shRNA) using GenBank as the basis. Initially, two sequences were designed to target MS4A4A, and a noninterfering sequence (sh‐NC) was used as a negative control. The primer sequences are presented in Table S1–S4. GenePharma® (Shanghai Hanheng Biotechnology Co., Ltd., CH) synthesizes these oligonucleotides. The lentiviral packaging system was created utilizing pLKO.1, a lentiviral gene silencing vector, CH, obtained from Hanheng Biotechnology Co., Ltd., Shanghai. The packaged virus and the target vector were transfected into 293T cells simultaneously using Lipofectamine 2000 when the cell confluence reached 80%–90%. After 48 h of cell culture, the supernatant was collected and then filtered and centrifuged to obtain the supernatant containing viral particles. Viruses should be collected during the exponential growth phase, and their viral titer should be determined through testing. Genechem (Shanghai Genechem Co., Ltd., China) constructed and packaged the lentivirus for gene overexpression. The lentiviral gene overexpression vector used was LV‐PDGFRA, which was developed by Hanheng Biotech Co., Ltd. (Shanghai, China).^31^, ^32^, ^33^, ^34^

### 2.11 Cell transfection and grouping

To construct a THP‐1 cell line overexpressing MS4A4A, the human monocytic cell line THP‐1 should be cultivated to around 50% cell density. Subsequently, it should be infected with lentivirus at a concentration of 10^6^ IU/mL, and the infection should be performed at a MOI of 0.8. Following infection, cells were treated with 10 μg/mL of puromycin (Sigma‐Aldrich, USA, catalog number 540222) for 48 h. Stable transfected cell lines were then maintained for at least 1 week for selection.

The cells are divided into groups 1, oe‐NC (cells infected with oe‐NC lentivirus), and 2, oe‐MS4A4A (cells infected with oe‐MS4A4A lentivirus). PMA (50 ng/mL) was subsequently employed to induce differentiation of engineered THP‐1 cells into M0 macrophages, followed by the stimulation of M0 macrophages to polarize into the M2 phenotype using IL‐4 (20 ng/mL). The expression levels of MS4A4A and M2 markers (CD163, VEGFA, IL‐10, ARG1, and TGFB1) were then quantified using qRT‐PCR.

To construct the MS4A4A knockdown BMDMs cell line, bone marrow cells were obtained from C57BL/6N mice using an in vitro method. Subsequently, these cells were induced to differentiate into BMDMs by employing an L929 cell‐conditioned medium (L929‐CM). Subsequently, on the sixth day, the knockdown of MS4A4A was achieved by transfecting BMDMs with either MS4A4A‐specific shRNA or control shRNA. Infect using a lentivirus with a titer of 10^6^ IU/mL and infect at a multiplicity of infection (MOI) of 0.8. Following a 48‐h infection period, macrophages were then cultured in a CM from CT2A or GL261 cells for an additional 24 h. Next, the selection was done by treating the cells with 10 μg/mL of puromycin (Sigma‐Aldrich, USA, 540222) for at least 1 week to identify the stably transfected cell lines.

The cells were divided into two groups: (1) the sh‐NC group, consisting of cells infected with sh‐NC lentivirus, and (2) the sh‐MS4A4A group, consisting of cells infected with sh‐MS4A4A lentivirus. To induce M2 polarization, the cells were coincubated with 20 ng/mL of recombinant mouse IL‐4 (peproTech, USA, AF‐214‐14) and 20 ng/mL of recombinant mouse IL‐13 (peproTech, USA, AF‐210‐13) for 48 h.^28^ During the TAM stimulation experiment, the BMDM cells were treated with CT2A/GL261 culture supernatant for 2 days to generate TAM.

To generate MS4A4A‐expressing J774A.1 and RAW264.7 cell lines, J774A.1 and RAW264.7 cells were cultured until reaching a 50%–60% cell density. Infect using a lentivirus with a titer of 10^6^ IU/mL and infect at a MOI of 0.8. The selection process was conducted 48 h after infection using 10 μg/mL of puromycin (Sigma‐Aldrich, USA, 540222) for at least 1 week to establish a stable transfected cell line. Once the J774A.1 and RAW264.7 cells overexpressing MS4A4A have reached the appropriate cell density, they should be transferred to the previously prepared CM derived from GBM cells. The cells should be then incubated for 24–48 h to simulate the conditions observed in TAMs within the TME. Expression of the markers was detected after 24–48 h.

The HEK‐293T cells were cultured in DMEM supplemented with 10% FBS, 10 μg/mL streptomycin, and 100 U/mL penicillin. The cells mentioned earlier were cultured in a humidified incubator (Thermo Fisher Scientific™, Germany, Heracell™ Vios 160i CR CO_2_ incubator) at 37°C and a CO_2_ concentration of 5%. Passage culture should be conducted when cell growth has reached 80–90%.^35^, ^36^

### 2.12 RT‐qPCR

RNA extraction was performed from each group of cells using TRIzol (15596026, Thermo Fisher Scientific, USA). The concentration and purity of the extracted RNA were determined using the NanoDrop 2000 spectrophotometer (ND‐2000, Thermo Fisher Scientific, USA). Next, mRNA was transcribed in reverse to cDNA using the PrimeScript RT reagent Kit (RR047A, Takara, Japan) per the instructions. RT‐qPCR detection was performed for the synthesized cDNA samples using the Fast SYBR Green PCR kit (11736059, Thermo Fisher Scientific, USA). Each sample was run in triplicate. In this experiment, β‐actin is employed as the internal reference gene for mRNA. The fold change in gene expression is determined by calculating 2^−ΔΔCt^, where ΔΔCT is calculated as ΔCt of the experimental group minus ΔCt of the control group, and ΔCt is calculated as Ct of the target gene minus Ct of the internal reference gene. In this context, Ct refers to the cycle number of amplifications needed for the real‐time fluorescence intensity of the reaction to reach the predetermined threshold value. It signifies the amplification process is in its logarithmic growth phase.^37^ The experiment was repeated three times. For primer sequence, refer to Table S2.

### 2.13 Western blot

The cells were lysed in RIPA lysis buffer (P0013B, Beyotime Biotechnology, China) to extract total proteins. After incubating on ice for 30 min, the lysates were centrifuged at 8000*g* for 10 min at 4°C. The resulting supernatant was collected. Protein concentration was quantified using the BCA assay kit (A53226, Thermo Fisher Scientific, USA). Following separation by polyacrylamide gel electrophoresis, the protein was transferred to a PVDF membrane (IPVH85R, Millipore, Darmstadt, Germany) using a wet transfer method. The sample should be incubated at room temperature in a solution containing 5% bovine serum albumin (BSA) for 1 h. The sample should be incubated overnight with the primary antibody at a temperature of 4°C (Table S3).

The membrane was washed three times with TBST, each time for 10 min. It was then incubated with HRP‐conjugated goat antirabbit IgG H&L secondary antibody (ab97051, diluted 1:2000, Abcam, Cambridge, UK) and goat antimouse antibody (ab205719, diluted 1:2000, Abcam, Cambridge, UK) for 1 h. After washing with TBST, place the membrane onto a clean glass slide. Prepare a mixture of Solution A and Solution B in appropriate proportions, taken from the ECL fluorescence detection kit (abs920, from ShangHai AbGenoBio), and carefully apply it onto the membrane in a darkened room. Subsequently, analyze the grayscale value of the target or β‐actin protein band using Quantity One V4.6.2 software provided by Bio‐Rad. This value indicates the relative protein content.^38^ The experiment should be repeated three times, and the average value should be calculated.

### 2.14 CCK‐8

#### 2.14.1 Flow cytometry for detecting cell apoptosis

In order to determine the role of T cells in cell apoptosis, Annexin V‐FITC cell apoptosis detection kit (C1062S, BioVision) was used to assess the apoptosis status of tumor cells. CT2A cells cocultured with T cells were seeded in a six‐well plate at a density of 5 × 10^5^ cells per well for culture and stained with FITC‐labeled Annexin V and propidium iodide (PI) for RA‐FLSs. After treatment, suspension cells and adherent cells were collected, washed with prechilled PBS, and then resuspended in 100 μL of binding buffer. To each tube, 10 μL of FITC‐labeled Annexin V and 5 μL of PI were added, followed by an incubation in the dark for 15 min. Subsequently, the samples were analyzed using a flow cytometer. Quadrant 1 (Q1) in the upper left represents cell debris with no cell membrane or dead cells for other reasons; Quadrant 2 (Q2) in the upper right indicates late‐stage apoptotic cells; Quadrant 3 (Q3) in the lower left represents normal (live) cells; Quadrant 4 (Q4) in the lower right indicates early‐stage apoptotic cells. Statistical analysis was performed on quadrants Q2 and Q4.^39^

### 2.15 CCK‐8

Cell proliferation experiments were statistically analyzed using the CCK‐8 assay kit. In the CCK‐8 assay, CT2A and GL261 tumor cells were treated with supernatants from mouse bone marrow‐derived TAMs containing sh‐NC or sh‐MS4A4A. Subsequently, cancer cells were seeded in 96‐well plates at a density of 2500 cells per well and treated with 10 μL of CCK‐8 reagent (96992, Sigma‐Aldrich, USA) at 0, 24, 48, 72, 96, and 120 h. The plates were then incubated at 37°C for 3 h, and the absorbance values at 450 nm wavelength for each well were measured on a microplate reader (M1000 PRO, Tecan). The absorbance values were directly proportional to the number of proliferating cells in the culture medium, and cell growth curves were constructed. The experiment was performed with five replicates and repeated twice.^40^

### 2.16 EdU experiment

CT2A and GL261 cells were treated with TAM supernatant containing sh‐NC or sh‐MS4A4A. The glioma cells were inoculated onto a 24‐well plate for testing. To the culture medium, add Guangzhou RiboBio Co., Ltd.'s EdU (C10310‐2) at a concentration of 10 μmol/L and incubate it in a culture chamber for 2 h. The medium was aspirated, and the cells were fixed with a 4% paraformaldehyde PBS solution at room temperature for 15 min. The samples were washed twice with PBS containing 3% BSA. Subsequently, they were incubated with PBS containing 0.5% Triton‐100 for 20 min at room temperature. Wash twice with PBS containing 3% BSA. Staining reagent (100 μL) was added to each well, followed by incubation at room temperature, in the absence of light, for 30 min. The nuclei were stained with DAPI for 5 min, then sealing the slides. Subsequently, 6–10 fields were randomly observed under Japan's Olympus fluorescence microscope (BX63), and the number of positive cells in each field was recorded. The EdU labeling rate is calculated by dividing the number of positive cells by the sum of the positive and negative cells and then multiplying the result by 100%.^41^ Repeat the experiment three times each.

### 2.17 Transwell assay

CT2A and GL261 cells were treated with TAM supernatant containing sh‐NC or sh‐MS4A4A. Thaw the Matrigel gel, which has been stored at −80°C, and allow it to equilibrate at 4°C overnight until it reaches a liquid state. Add 200 μL of Matrigel to 200 μL of serum‐free medium at 4°C, and mix thoroughly to dilute the gelatinous substance. Next, add 50 μL to each Transwell plate, transfer them to the incubator, and incubate for 2–3 h until the gel is fully solidified. The cells should be digested and counted before preparing a cell suspension in a serum‐free culture medium. Place 200 μL of cell suspension into each well of the upper chamber and add 800 μL of culture medium supplemented with 20% FBS to the lower chamber. Incubate for 24 h in a 37°C incubator. The Transwell plate was removed and rinsed twice with PBS, the cells on the upper surface were wiped off using a cotton ball, and it was fixed with formalin for 10 min, followed by three washes with water. The specimen should be stained with a 0.1% crystal violet solution and left at room temperature for 30 min. Afterward, it should be rinsed twice with PBS. Observe, take photos, and count under an inverted microscope. Migration experiments do not require gel coating application to the substrate, and the incubation duration is set at 24 h. Count at least four randomly selected areas of cells under the microscope.^42^, ^43^

### 2.18 Scratch experiment

CT2A and GL261 cells were treated with TAM supernatant containing sh‐NC or sh‐MS4A4A. The processed test cells should be seeded separately in six‐well plates at a cell density ranging from 70% to 90%. A clear and visible scratch can be generated by using a 200‐μL pipette tip to be gently drawn across the surface of the well plate. Clean the cells to remove any detached ones and add fresh culture medium. The samples were incubated at 37°C with 5% CO_2_. The initial condition of scratches was recorded at 0 h, and observations and photographs were taken using Japan's Olympus inverted microscope (CKX53). After 24 h, the condition of the scratches should be reevaluated using an inverted microscope for observation and photography. The width of scratches was measured using image processing software, specifically ImageJ, and the distance of cell migration was subsequently calculated.^44^

### 2.19 Iron and reactive oxygen determination

The Invitrogen™ Cell Total Iron Colorimetric Assay Kit (EEA009, USA) should be used to measure the total iron levels in TAM cells, following the manufacturer's instructions. The cells were rapidly and uniformly suspended in an experimental iron buffer. Subsequently, centrifugation at 16,000 *g* for 10 min at 4°C was performed to eliminate insoluble substances. Add approximately 50 μL of the sample to each well of a 96‐well plate. Adjust the sample volume to 100 μL by adding 50 μL of buffer solution. Next, add approximately 5 μL of either iron determination buffer or iron‐reducing agent separately to determine the concentration of ferrous iron or total iron. After mixing, incubate the mixture in the dark at 25°C for 30 min. Then, add 100 μL of iron probe to each well and continue incubating in the dark at 25°C for 60 min. Measure the absorbance at 550 nm and then calculate the concentration of iron using the standard curve. Following the manufacturer's instructions, cells were treated with Invitrogen's oxidation sensitive probe, 2′,7′‐dichlorodihydrofluorescein diacetate (CM‐H2DCFDA, C6827, USA), at 37°C for 10 min to quantify the intracellular levels of reactive oxygen species (ROS).^45^

### 2.20 Macrophage inhibition function test

The successful collection and separation of bone marrow‐derived macrophages (BMDMs) from C57BL/6NN mice was accomplished. Likewise, mouse spleens were washed with PBS, and single‐cell suspensions were obtained by passing the spleen through a 70‐μm cell strainer. Mix CT2A or GL261 cells with splenocytes and BMDMs in a ratio of 1:1:1. The cells were cultured on a plate coated with 5 μg/mL of antimouse CD3ε (145‐2C11, Biolegend, USA) and 1 μg/mL of soluble antimouse CD28 (BE0015, Bio X Cell, USA). They were treated with 10 μg/mL of MS4A4A antibody for 2 days. Cells are collected after cultivation and analyzed using the CytoFlex flow cytometer from Beckman Coulter. To begin, CD8^+^ T cells are initially sorted using flow cytometry with antibodies labeled with CD3 and CD8. Subsequently, the expression of Ki‐67 is measured and quantified via flow cytometry.^28^

### 2.21 In vitro macrophage induction experiment

To induce differentiation into M0 macrophages, THP‐1 cells were incubated with 50 ng/mL of phorbol 12‐myristate 13‐acetate (PMA) from InvivoGen (USA) for 48 h. To further polarize M1, cells were incubated with 100 ng/mL lipopolysaccharide (LPS, L2630, Sigma‐Aldrich, USA) and 20 ng/mL recombinant human interferon‐γ (Recombinant Human IFN‐γ, AF‐300‐02, PeproTech, USA) for 48 h. As a control experiment, we conducted the same incubation treatment in PBMC cells (PCS‐800‐011, ATCC, USA) as in THP‐1 cells. To induce M2 polarization, we incubated the samples with 20 ng/mL of recombinant human interleukin‐4 (Recombinant Human IL‐4, AF‐200‐04, peproTech, USA) for 48 h. A similar strategy was employed to induce the differentiation of BMDMs. To achieve M1 polarization, cells were incubated for 48 h with 100 ng/mL of LPS and 20 ng/mL of recombinant murine interferon γ (Recombinant Murine IFN‐γ [AF‐315‐05, peproTech, USA]). To induce M2 polarization, the cells were incubated with 20 ng/mL of recombinant murine interleukin‐4 (Recombinant Murine IL‐4, AF‐214‐14, PeproTech, USA) and 20 ng/mL of recombinant murine interleukin‐13 (AF‐210‐13, PeproTech, USA) for 48 h.^28^ Establish an untreated control group, called the “Blank” group, in which no lentivirus transfection or addition of interleukins (IL‐4/IL‐13) is performed to induce M2 polarization. This control group demonstrates the baseline expression levels of cells in their natural state.

### 2.22 CRISPR/Cas9

The MS4A4A knockout (MS4A4A^−/−^) mouse line was created using CRISPR/Cas9‐mediated gene editing methodology on a C57BL/6NN background. The mice were obtained from GemPharmatech (Jiangsu JiCui Pharmaceutical Technology Co., Ltd., China). Procure 6‐week‐old C57BL/6NN mice weighing 25–30 g from Beijing Vitalstar Biotechnology Co., Ltd. (213, Vitalstar, CH). The mice were kept in SPF‐grade animal facilities, where the humidity was maintained at 60–65%, and the temperature was controlled at 22–25°C. When subjected to alternating periods of 12 h of light and darkness, mice should be provided with sufficient food and water. This study utilized both male and female mice, and their gender was not considered as a covariate. The mice are housed in a controlled environment with a 20–26°C temperature and a 30%–70% humidity level. A 12‐h light–dark cycle is maintained. The experiment commenced 1 week after the adaptive feeding period, and the health status of the mice was assessed before the experiment. All animal research is conducted in accordance with the institute's “Guidelines for the Care and Use of Laboratory Animals.”^46^

### 2.23 Construct a mouse GBM model

A mouse GBM model was established by injecting CT2A and GL261 glioma cells subcutaneously or intracranially into mice. Each mouse was injected with a cell suspension containing 1 × 10^5^ CT2A tumor cells in 1–5 μL for intracranial injection. The injection site was approximately 2 mm posterior to the nose bridge, 2 mm lateral to the midline, and at a depth of approximately 3 mm. For subcutaneous injection, each mouse was administered with a cell suspension containing 2.5 × 10^6^ CT2A or 3 × 10^6^ GL261 tumor cells in a volume of 100–200 μL. Both models were injected on day 1, and tumor growth and progression were observed daily afterward. Tumor volume was measured on days 3, 6, 12, 24, and 30. Our study did not exceed the maximum tumor volume limit of 1500 mm^3^ set by the Research Ethics Committee. The experiments described before aimed to model different types of tumor cells. A total of 12 mice were randomly assigned into two groups, each consisting of six mice: (1) the WT group, which included wild‐type mice inoculated with either CT2A or GL261 tumor cells, and (2) the MS4A4A^−/−^ group, comprising MS4A4A knockout mice inoculated with either CT2A or GL261 tumor cells.^46^

In this experiment, the phagocytosis of mouse macrophages was studied. Intraperitoneal injections of Clodronate liposomes were administered to efficiently deplete TAMs in mice. Each mouse received 150 μL of Clodronate liposomes (C419188‐2, Aladdin, CH) and control liposomes (PBS) (C419188‐2, Aladdin, CH) intra‐abdominally. After the injection, mice should be observed for a minimum of 30 min to ensure the absence of any adverse reactions. The specific treatment procedure is as follows: on day 0, an injection of CT2A or GL261 tumor cells is administered. On the day before and days 3, 7, 10, 16, and 17 following the injection of tumor cells, administer intraperitoneal injections of 150 μL of Clodrosome and PBS. A weight and health assessment should be performed before each experiment. Following the experiment, the efficacy of chlorphosphonic acid liposomes in depleting TAMs was evaluated through necessary biomarker, tissue, and cytological analysis.

The earlier experiments were conducted to model different tumor cells. Mice were randomly divided into four groups, each consisting of six mice: (1) WT + CL group, which included wild‐type mice injected with either CT2A or GL261 tumor cells and 150 μL of Clodrosome; (2) WT + PL group, which included wild‐type mice injected with either CT2A or GL261 tumor cells and 150 μL of PBS; (3) MS4A4A^−/−^ + CL group, which included MS4A4A^−/−^ mice injected with either CT2A or GL261 tumor cells and 150 μL of Clodrosome; (4) MS4A4A^−/−^ + PL group, which included MS4A4A^−/−^ mice injected with either CT2A or GL261 tumor cells and 150 μL of PBS.^28^

### 2.24 Identification of MS4A4A gene knockout

#### 2.24.1 Peripheral blood collection

Blood was collected from the tail vein of mice using microtubes containing the anticoagulant EDTA (Sigma‐Aldrich, USA). Cell separation was performed by density gradient centrifugation to obtain a single nuclear cell layer, which included white blood cells. The individual cell layer should be collected into a new microcentrifuge tube and washed with PBS at least twice. For cell lysis and protein extraction, RIPA buffer from Beyotime Biotechnology (China) should be used to ensure complete lysis using freeze–thaw, sonication, or filtration methods. After centrifugation to remove cell debris, the supernatant should be collected.

#### 2.24.2 Western blot verification

Protein concentration was quantified using the BCA assay kit (Thermo Fisher Scientific, USA). Following protein separation through polyacrylamide gel electrophoresis, the gel was wet transferred onto PVDF membranes (Millipore, Darmstadt, Germany). The specimens were incubated at room temperature with a 5% BSA solution for 1 h, then subjected to overnight incubation at 4°C with a primary antibody targeting MS4A4A (ABcIonaI, China). The membrane was washed three times with TBST (10 min each time) and then incubated with a 1:2000 diluted antimouse antibody (goat antimouse antibody from Abcam, Cambridge, UK) for 1 h. Following rinsing with TBST, carefully position the membrane onto a glass plate. Prepare a mixture of the appropriate amounts of ECL fluorescence detection reagents (obtained from Abcam [Shanghai] Bio‐technology Co., Ltd., Shanghai, China), consisting of Solution A and Solution B. Subsequently, apply the mixture onto the membrane in a dark room. Analyze the results using Quantity One V4.6.2 software from Bio‐Rad (USA) to determine the relative protein content, expressed as the grayscale value of the corresponding protein band concerning the β‐actin protein band.^38^ Five mouse samples should be taken per group. The detection should be repeated three times for each mouse sample, and the average value should be calculated.

### 2.25 Immune checkpoint blockade (ICB) therapy

Intracranial tumors were induced in wild‐type (WT) or MS4A4A^−/−^ mice through intracranial inoculation of 1 × 10^5^ CT2A cells. On day 6, the mice were intraperitoneally injected with 200 μg of anti‐PD‐1 antibody (29F.1A12, BioLegend, USA) or 100 μg of anti‐PD‐L1 antibody (10F.9G2, BioLegend, USA), followed by repeated injections every 3 days. Subcutaneous tumors were induced by intracranially implanting 1 × 10^5^ CT2A cells into wild‐type (WT) or MS4A4A^−/−^ mice. Anti‐PD‐1 antibodies were injected intraperitoneally at a dosage of 50 μg per mouse starting on day 12 and repeated every 4 days after that. These experimental protocols were employed to establish a murine model of GBM and implement ICB therapy. Every other day, data measurements on the mice were conducted to ensure the stability of their body weight and serum parameters. The specific reference range for body weight should not be exceeded by 10% of the initial weight, while serum proteins should be within the range of 35–55 g/L. The ranges for AST and ALT levels should be between 10 and 40 IU/L. All experiments were conducted in accordance with ethical and legal regulations, following the guidelines set by the Animal Ethics Committee.^46^

The experiments described earlier involved modeling different immunotherapies. Mice were randomly divided into four groups, with six mice in each group. The groups included: (1) WT group, which consisted of wild‐type mice inoculated with intracranial or subcutaneous CT2A tumor cells; (2) MS4A4A^−/−^ group, which consisted of MS4A4A^−/−^ mice inoculated with intracranial or subcutaneous CT2A tumor cells; (3) WT + anti‐PD‐1/anti‐PD‐L1 group, which consisted of WT mice inoculated with intracranial or subcutaneous CT2A tumor cells and injected with 200 μg of anti‐PD‐1/PD‐L1 antibodies each; (4) MS4A4A^−/−^ + anti‐PD‐1/anti‐PD‐L1 group, which consisted of MS4A4A^−/−^ mice inoculated with intracranial or subcutaneous CT2A tumor cells and injected with 200 μg of anti‐PD‐1/PD‐L1 antibodies each.^28^

### 2.26 Tumor‐infiltrating cells (TICs)

To prepare single‐cell suspensions, we harvested the spleens and tumor tissues from WT mice and MS4A4A^−/−^ mice, which had previously received subcutaneous injections of CT2A glioma cells. Subsequently, these tissues were cultured in a DMEM supplemented with 10% fetal bovine serum (FBS). The tumor tissue was dissected and collected in a DMEM containing 0.5 mg/mL of collagenase IV (c4‐22‐1G, Sigma‐Aldrich, USA) and 0.1 mg/mL of DNase I (D5025, Sigma‐Aldrich, USA). It was followed by incubating the samples on a shaker at 37°C for 1 h. Subsequently, mechanical dispersion was carried out on glass slides coated with sandpaper. The spleen is mechanically separated directly onto a frosted glass slide. Tumors or suspensions of splenocytes were passed through a 70‐μm cell strainer (F8210, Solarbio Life Science, CH) to obtain single‐cell suspensions. The red blood cells were lysed by 2 mL of RBC lysis buffer (R7757, Sigma‐Aldrich) at room temperature for 2 min. The lysis was terminated by adding DMEM. The single‐cell suspension was quantified and then 1 × 10^6^ cells were plated in a 24‐well plate for subsequent analysis of the composition of immune cells. In summary, our experiment utilized multicolor flow cytometry to sort distinct immune cell subpopulations from mouse tissue samples.

To begin with, all the cells are labeled with PE‐CD45 to facilitate the identification and sorting of white blood cells. We sorted specific subsets of cells based on different cell surface markers. Specifically, we used the following antibody combinations: (1) To sort natural killer (NK) cells, we used APC‐CD3 and PerCP‐Cy5.5‐NK1.1. (2) To sort B cells, we used APC‐CD3 and PE‐Cy7‐CD19. (3) To sort CD4^+^ T cells, we used APC‐CD3 and FITC‐CD4. (4) To sort CD8^+^ T cells, we used APC‐CD3 and PE‐CD8.

To sort different types of myeloid cells, the PE‐CD45 antibody is initially used to label and identify leukocytes, followed by employing a specific antibody combination to sort the target cell subsets. Cells are labeled with PE‐CD45 to sort macrophages and subsequently sorted using BV421‐Ly6G, BV510‐CD11b, and PE‐F4/80 antibodies. For sorting monocytes, cells are similarly labeled with PE‐CD45 and sorted using BV510‐CD11b, BV421‐Ly6G, and PE‐Cy7‐Ly6C antibodies. Antibody sorting was performed using the CytoFlex flow cytometer (Beckman Coulter, Inc.) to achieve highly purified cell subpopulations.

We supplemented the cell culture medium with a cell activation mix (423303, BioLegend, USA) containing Brefeldin A for cytokine analysis. We used them for subsequent staining after stimulating lymphocytes for 4–6 h. The phenotypic and functional analyses were conducted on the aforementioned grouped cells. First, we examined the expression of IFN‐γ in CD8^+^ T cells, which were labeled with BV421‐IFN‐γ antibody to evaluate their functional status in immune response. Additionally, the expression of Ki67 in CD8^+^ T cells was assessed using the BV421‐Ki67 antibody to determine their proliferative activity.

Moreover, the expression of PD‐1 in CD8^+^ T cells was examined by labeling them with PE‐Cy7‐PD‐1 antibody to evaluate their immune checkpoint status. Finally, we investigated the expression of CD206 in macrophages by labeling them with APC‐CD206 antibodies to assess their M2 polarization status. All the above experiments were conducted using the CytoFlex flow cytometer (Beckman Coulter, Inc.).^28^

### 2.27 Flow cytometry

To perform surface staining, add the recommended amount of surface antigen–antibody as specified in the antibody manual and incubate for 30 min. As mentioned, cells are subjected to surface antigen staining for intracellular cytokine analysis. Next, the cells were incubated with Staining Buffer (420201, Biolegend, USA) at room temperature for 20 min. Next, the fixed cells were resuspended in the Intracellular Staining Permeabilization Wash Buffer (10X) (421002, Biolegend, USA) and continue with intracellular cytokine staining. To perform nuclear protein staining, resuspend the cells in 1× FOXP3 Fix/Perm Buffer (421403, Biolegend, USA), ensuring thorough mixing, and then incubate at room temperature for 20 min. Resuspend the cells in 1× FOXP3 Fix/Perm buffer and incubate them at room temperature for 15 min. Centrifuge the solution to remove the supernatant and then resuspend the cells in 1× FOXP3 Perm buffer. After that, proceed with nuclear protein staining. All flow cytometry experiments were conducted using the CytoFlex flow cytometer (Beckman Coulter, Inc.). Table S4 lists all the antibodies employed in the flow cytometry analysis.^28^

### 2.28 ELISA assay

ELISA was performed according to the manufacturer's instructions. The cells should be seeded at an appropriate concentration into a 96‐well plate and cultured for 24 h. Next, the cell culture medium should be removed and replaced with an equivalent volume of DMEM free of serum. The supernatant should be collected after 24 h and passed through a 0.45‐μm filter to eliminate any suspended cells. The levels of secreted interleukin‐10 (IL‐10, 900‐T53, Invitrogen) and transforming growth factor (TGF)‐β protein (TGF beta‐1, BMS608‐4, Invitrogen) in the cell culture supernatant were measured using the Mouse ELISA Kit.^28^

### 2.29 Histological staining

Hematoxylin and eosin (H&E) staining was performed on subcutaneous transplant tumor samples obtained from mice. The samples were fixed and sectioned, followed by the removal of the paraffin blocks. Xylene was employed for paraffin removal. Subsequently, the sections were dehydrated sequentially in 100%, 95%, and 70% ethanol solutions. Finally, the sections were either mounted or washed with water. The prepared slices are placed in the Sudan black staining solution (H8070, Solarbio, China) and then stained at room temperature for 5–10 min. Next, rinse the slices with distilled water and dehydrate them in 95% ethanol. Subsequently, immerse the slices in a staining solution (G1100, Solarbio, China) for 5–10 min. Finally, carry out routine dehydration, clearing, and mounting.^47^

### 2.30 Immunohistochemical staining

Tumor samples were collected from patients with glioma who underwent surgical procedures. The samples were fixed in 4% paraformaldehyde overnight and then embedded in paraffin with a thickness of 4 μm. Deparaffinization was achieved using xylene, with subsequent alcohol dehydration using absolute ethanol, 95% ethanol, and 75% ethanol, each for 3 min. The sample should be boiled in 0.01 M citrate buffer for 15–20 min for antigen retrieval. Afterward, incubate it at room temperature in 3% H_2_O_2_ for 30 min to inactivate endogenous peroxidase. Next, block it with a goat serum‐blocking solution and allow it to sit at room temperature for 20 min before removing any excess liquid.

Add the corresponding primary antibody according to Table S3 and incubate at room temperature for 1 h. Afterward, wash with PBS and introduce the secondary antibody, IgG (ab6785, 1:1000, Abcam). Incubate at 37°C for 20 min, followed by another wash with PBS. Subsequently, add the SP (Streptavidin–HRP) and incubate at 37°C for 30 min. Following a wash with PBS, perform color development using DAB (P0202, Beyotime Biotechnology Co., Ltd.) for 5–10 min. Finally, wash with water for 10 min to halt the reaction. Xylene (C0107, Beyotime Biotechnology Co., Ltd.) was used for 2 min for restaining. Differentiation was performed using hydrochloric acid alcohol and a 10‐min water wash. Dehydration was carried out with gradient alcohol (transparent with xylene), and finally, 2–3 drops of neutral resin were added for mounting.

To collect data, randomly select five high‐power magnification fields of view on each slide and then observe and count under a brightfield microscope. The same procedure was followed for the immunohistochemical staining of tumor tissues transplanted subcutaneously in mice. Staining intensity is classified into four levels based on overall assessment: 0 (absent), 1 (weak), 2 (moderate), and 3 (strong). The percentage of cancer cells stained positively is divided into three levels: ≤10% (1 point), >10% to ≤50% (2 points), and >50% (3 points). The final score is calculated by multiplying the intensity score with the percentage score for each case (intensity score × percentage score). The resulting score ranges from 0 to 9. A final score of ≤1 indicates a negative expression, while ≥2 is considered a positive expression.^48^

### 2.31 Statistical analysis

The data were obtained from at least three independent experiments and are presented as the mean ± standard deviation (SD). To compare the two groups, an independent samples *t* test is utilized. For comparing three or more groups, analysis of variance (ANOVA) is utilized. If the ANOVA yields differences, we will conduct Tukey's HSD test to compare the differences among groups. We will employ either the Mann–Whitney *U* test or the Kruskal–Wallis *H* test to examine non‐normal distribution or heteroscedastic data. The Seurat package was utilized to process and analyze single‐cell transcriptome data. We conduct cell clustering by utilizing gene expression profiles and then visualize the results using UMAP. Utilize the FindAllMarkers function to identify DEGs and apply an adjusted *p*‐value threshold of <0.05 to establish statistical differences. We conducted pathway enrichment analysis using the clusterProfiler package, focusing on DEGs. Statistical analyses were conducted using GraphPad Prism 9 (GraphPad Software, Inc.) and the R language. The significance level for all tests is set at 0.05, and any two‐sided *p* value below 0.05 is regarded as statistically significant.

## 3 RESULTS

### 3.1 Single‐cell RNA‐Seq analysis reveals differential macrophage distribution across GBM stages and its potential role in disease progression and treatment response

**FIGURE 1:**
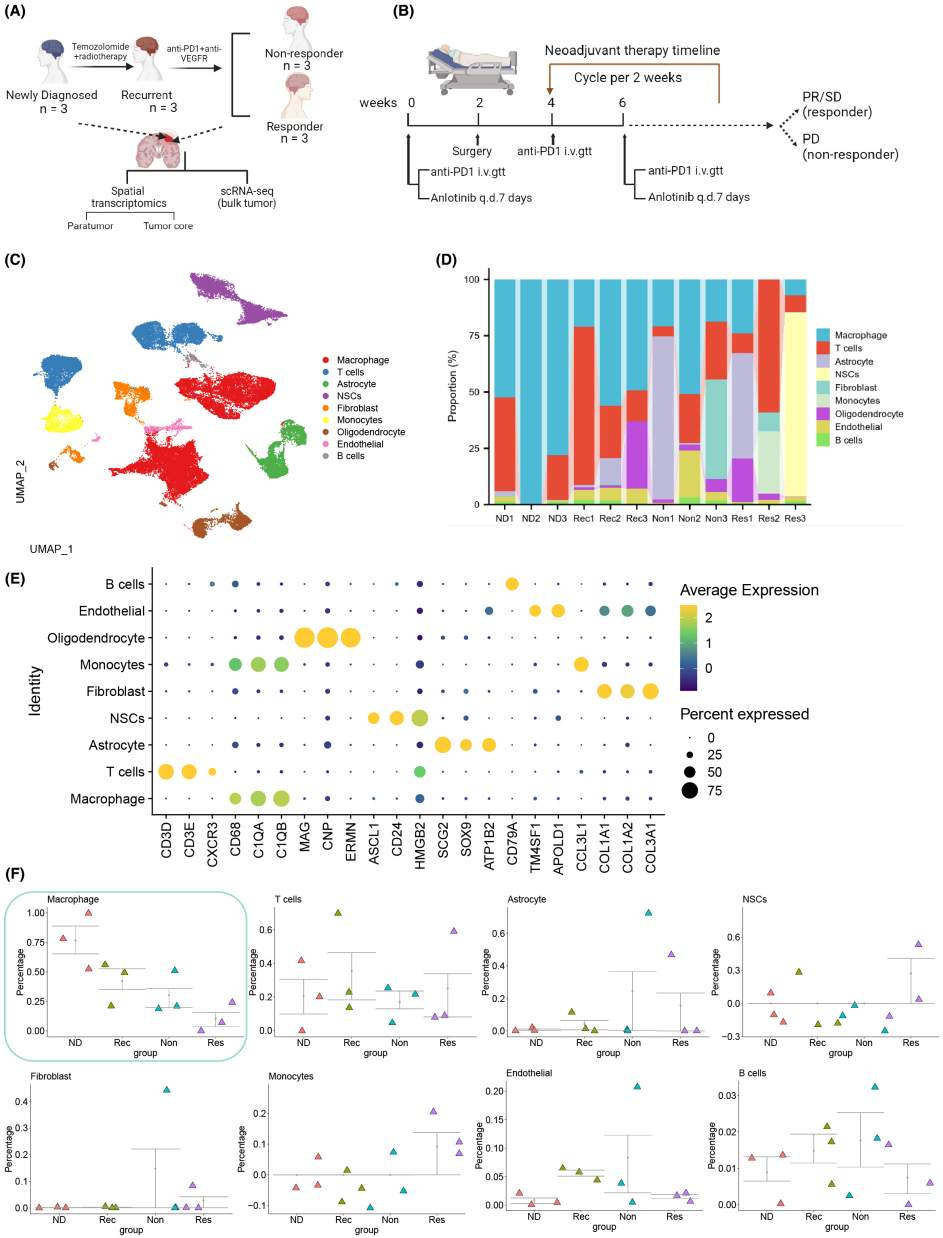
Molecular characterization differences in glioma patients at different treatment stages. (A) Experimental workflow for scRNA‐seq and ST data analysis of GSE235676 dataset. (B) Illustration of PD‐1 adjuvant therapy in the GSE235676 dataset. (C) TSNE clustering analysis of cells into nine cell clusters. (D) Proportional distribution of different cell subtypes in each sample, represented by different colors (*n* = 9). (E) UMAP expression maps of specific marker genes for each of the nine cell clusters. (F) Changes in the proportional distribution of different cell subtypes across different treatment stages, represented by different colors (*n* = 9).

In this study, the GSE235676 dataset, which contains single‐cell RNA sequencing (scRNA‐seq) data from four types of GBM patients, was obtained from the GEO database (http://www.ncbi.nlm.nih.gov/geo/). The dataset comprises four groups: seven cases of newly diagnosed GBM patients (ND), three cases of patients with recurrent GBM who have undergone temozolomide (TMZ) and radiation therapy (Rec), three cases of patients with no response to pembrolizumab (PD‐1 inhibitor) and alectinib combination therapy (Non), and three cases of patients with a positive response to pembrolizumab (PD‐1 inhibitor) and alectinib combination therapy (Res) (Figure 1A,B).

scRNA‐seq analysis was conducted on the data above sample, and the sequencing data were integrated using the Seurat package. Initially, we assessed the number of genes (nFeature_RNA), mRNA molecules (nCount_RNA), and the proportion of mitochondrial genes (percent.mt) in all cells of the scRNA‐seq data. The results revealed that most cells exhibited nFeature_RNA < 5000, nCount_RNA < 20,000, and percent.mt < 5% (Figure S1A).

We conducted data quality control by applying the 200 < nFeature_RNA < 5000 and percent criteria.mt < 5. Following removing low‐quality cells and duplicate genes, we obtained an expression matrix comprising 57,854 genes and 27,268 cells. The correlation analysis of sequencing depth revealed that the correlation coefficient (*r*) between the filtered data nCount_RNA and percent.mt was −0.58, and the correlation coefficient (*r*) between nCount_RNA and nFeature_RNA was 0.99 (Figure S1B). These results substantiate that the filtered cell data are of good quality and suitable for subsequent analysis.

Further analysis was conducted on the filtered cells, where genes with high variability in gene expression were selected based on variance. The top 2000 genes with the highest variability, as determined by variance, were chosen for subsequent analysis (Figure S1C). Next, the dataset was subjected to linear reduction using PCA with the selected highly variable genes, and the resulting PCs were visualized in a plot (Figure S1D). This study presents the primary correlation heatmap of gene expression for PC_1–PC_4 (Figure S1E). Additionally, ElbowPlot is employed to sort PCs based on their standard deviation (Figure S1F). The results indicate that PC_1–PC_4 effectively reflect the information conveyed by the chosen height‐variable genes and demonstrate analytical importance.

Next, we apply the UMAP algorithm to perform nonlinear dimensionality reduction on the first 15 PCs, using a resolution of 0.2 for cluster analysis (Figure S1G). We obtained 21 clusters through cluster analysis and the clustering information for each group (Figure S2A–C). We identified and annotated known lineage‐specific marker genes for cells using the online database CellMarker (Figure 1C; Figure S2D) through a literature search. The expression pattern of marker genes for each cell type was analyzed (Figure 1E), identifying nine cell categories. Clusters 0, 1, and 3 were identified as macrophages; clusters 2, 7, 9, and 17 as T cells; clusters 4 and 13 as astrocytes; clusters 11, 12, 18, and 20 as oligodendrocytes; cluster 16 as B cells; clusters 14 and 19 as endothelial cells; cluster 10 as monocytes; and clusters 8 and 15 as fibroblasts. Additionally, clusters 5 and 6 were identified as neural stem cells (NSCs).

Significant differences in the number of macrophages were observed in the tissues of recurrent patients (Rec) and patients treated with PD‐1‐1 combination therapy (Non and Res) compared to the samples of newly diagnosed (untreated) patients (ND). Among all cells, the proportion of macrophages reached 39.49%. Furthermore, a gradual decrease in macrophages was observed during the progression of the disease stages (ND → Rec → Non → Res), suggesting the potential importance of macrophages in the progression and treatment of GBM (Figure 1D,F). The annotation of macrophages was validated (Figure S2E–G).

The results before indicate that GBM samples from the four diagnostic stages could be classified into 21 clusters, comprising nine cell subgroups. It was observed that the number of macrophages decreases gradually as the disease progresses, highlighting the role of macrophages in the progression and treatment of GBM.

### 3.2 MS4A4A overexpression in TAMs correlates with poor response to PD‐1 immunotherapy and adverse prognosis in GBM patients

**FIGURE 2:**
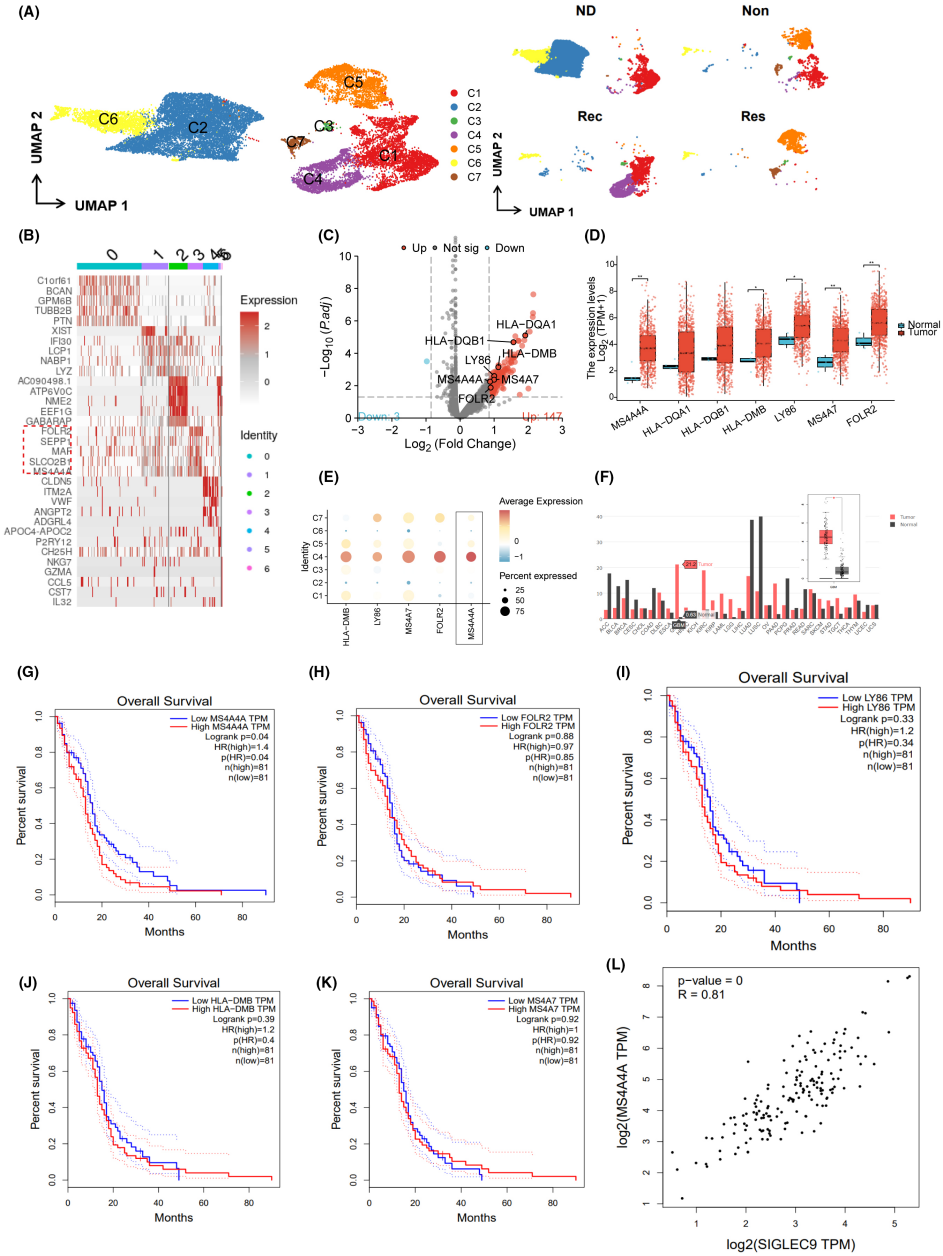
High expression of MS4A4A in tumor‐associated macrophages and its correlation with poor prognosis in cancer patients. (A) Detailed classification and distribution trajectory of macrophage clusters in GBM (*n* = 3). (B) Heatmap showing the expression of marker genes in different cell clusters, with red indicating upregulation and white indicating downregulation (*n* = 3). (C) Volcano plot showing differentially expressed genes between the Non and Res groups of the C4 cell cluster (*n* = 3). (D) Differential gene expression between normal and glioma tissues according to TCGA data. (E) Bubble plot showing the expression of differentially expressed genes in different macrophage clusters. (F) Analysis of MS4A4A gene expression in various tumors using the GEPIA database; (G–K) The GEPIA database to analyze the prognostic situation of differential genes in GBM, categorizing samples into two groups based on the median gene expression level (50th percentile): high‐expression group (samples with all expression levels higher than the median) and low‐expression group (samples with all expression levels lower than the median). (L) Analysis of the correlation between MS4A4A and the immune checkpoint SIGLEC9 using the GEPIA database. *p < 0.05 compared to the normal group.

We reclustered the macrophages and identified seven distinct cell clusters, designated as C1–C7, for further analysis (Figure 2A,B). Notably, we observed an enrichment of SEPP1, MRC1, FOLR2, and MS4A4A in C4 macrophage clusters. Previous studies have demonstrated that SEPP1^+^ FOLR2^+^ macrophages, also known as TAMs, play a role in promoting both GBM recurrence and inflammation.^49^

MRC1 is a well‐known marker gene for M2 macrophages, while MS4A4A is thought to have a role in macrophage function and polarization, particularly in the expression of M2 macrophages that are known to promote tumor development in GBM.^50^ Hence, we classified the C4 cell cluster as M2 macrophages.

Prior studies have indicated that M2 macrophages are commonly linked to immune suppression, and the polarization state of these macrophages may influence the efficacy of immune suppressive signals. Therefore, we hypothesize that C4 cell clusters constitute the pivotal cell clusters in glioma patients who resist PD‐1 therapy.^51^, ^52^

We conducted differential analysis on the gene expression profiles in the C4 cell cluster between the Non and Res groups. We identified 147 upregulated genes and 3 downregulated genes. Using the FindAllMarkers function in the Seurat package, we also identified 56 marker genes with upregulated expression in the C4 cell cluster (Figure 2C). The results revealed upregulation of HLA‐DQA1, HLA‐DQB1, HLA‐DMB, LY86, MS4A7, FOLR2, and MS4A4A in the C4 cell cluster of the Non group.

To establish the correlation between these genes and the ineffectiveness of PD‐1 blockade therapy in GBM patients, we initially conducted a differential expression analysis on the seven key genes using GBM transcriptomic data from the TCGA database. The results indicated an increase in MS4A4A, HLA‐DMB, LY86, MS4A7, and FOLR2 expression in GBM tumor samples compared to normal tissue samples (*n* = 706) (Figure 2D).

Based on the single‐cell data analysis, the expression difference of MS4A4A in the C4 cell cluster was the most compared to other macrophage clusters (Figure 2E). Therefore, we hypothesize that MS4A4A could be a crucial factor contributing to patients' insufficient response to PD‐1 blockade therapy. Subsequently, we analyzed MS4A4A expression in various cancers using the GEPIA database. The findings aligned with previous research, demonstrating a noteworthy increase in MS4A4A expression in GBM (Figure 2F).

To investigate the impact of DEGs on the prognosis of glioma patients, we conducted a survival analysis using the GEPIA database. The genes analyzed included HLA‐DQA1, HLA‐DQB1, HLA‐DMB, LY86, MS4A7, FOLR2, and MS4A4A in glioma patients (Figure 2G–K). The analysis revealed a difference in the survival curve, specifically when comparing the high‐expression group (*n* = 81) and the low‐expression group (*n* = 81) of MS4A4A (Logrank *p* = 0.04). Higher expression of MS4A4A was associated with an increased risk of patient survival and showed a difference compared to the low‐expression group (*p*(HR) = 0.04). These findings indicate that high expression of MS4A4A is associated with poor survival outcomes in glioma patients (Figure 2G).

Research has shown that in animal models inhibition of MS4A4A or the use of anti‐MS4A4A monoclonal antibodies as therapy could modify the immune microenvironment of the tumor. This modification leads to a decrease in the infiltration of M2 macrophages and severely exhausted T cells while enhancing the infiltration of effector CD8^+^ T cells. These changes result in the inhibition of colorectal cancer growth.^28^

Recent research findings show that Siglec‐9 functions as an immune checkpoint molecule on macrophages in GBM.^29^ Our analysis of the GEPIA database revealed a positive correlation between the expression of MS4A4A and Siglec‐9 (Figure 2L).

In conclusion, MS4A4A exhibits high expression in the GBM‐associated macrophages of glioma patients resistant to PD‐1 immunotherapy, and is correlated with poor prognosis.

### 3.3 Role of MS4A4A in macrophage differentiation: implications for tumor growth and immune evasion across various cancers

**FIGURE 3:**
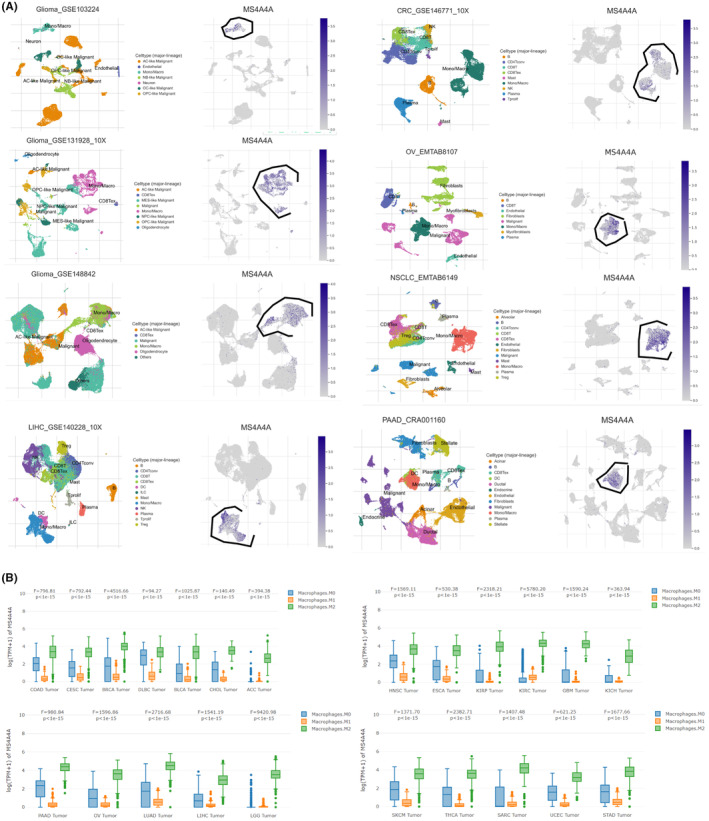
Analysis of MS4A4A expression in various cancer‐associated macrophages. (A) Analysis of MS4A4A gene expression in various tumors using the pan‐cancer scRNA‐seq dataset from the TISCH database (http://tisch.comp‐genomics.org/). (B) Analysis of MS4A4A expression in M2 macrophages in various tumors using the GEPIA2021 online tool.

Macrophages are crucial in the TME, and the MS4A4A gene is a vital regulator. To conduct a comprehensive analysis, we performed a series of studies to gain a deeper understanding of the expression patterns of MS4A4A in various types of cancer and its significance in macrophage development and function. After analyzing the TISCH database (http://tisch.comp‐genomics.org/), we discovered that the MS4A4A gene demonstrates specific expression in macrophages across different types of tumors (Figure 3A). The online tool GEPIA2021 (http://gepia2021.cancer‐pku.cn/) showed a substantial increase in MS4A4A gene expression in M2 macrophages of different tumor types (Figure 3B).

We conducted a pseudotime analysis to monitor the state transitions of macrophages throughout development, differentiation, or other continuous processes. The findings revealed that the early‐stage macrophages predominantly comprised C2 and C6 cell clusters, whereas C4 exhibited enrichment in the mid–late stages of macrophage differentiation (Figure S3A).

To identify the critical genes involved, we simulated the trajectories of three representative genes (MS4A4A, CD163, CD86) and observed that MS4A4A exhibited high activity in the later stage. The expression of CD163 initially increased and then reached a plateau, indicating the stabilization of the protumor growth effect of M2 macrophages in the later stages. Conversely, the expression of CD86 initially increased, but subsequently decreased, suggesting a weakened antitumor effect of M1 macrophages in the later stages (Figure S3B,C). Based on the findings before, we hypothesize that the elevated MS4A4A activity contributes to tumor evasion by involving itself in the differentiation of M1 and M2 macrophages.

The results before indicate that MS4A4A is markedly overexpressed in TAMs across different cancers, including GBM. Further temporal analysis indicates that MS4A4A may play a role in differentiating and functionally regulating M1 and M2 macrophages, consequently impacting tumor growth and development.

### 3.4 Spatial transcriptomics reveals distinct MS4A4A‐positive macrophage distribution in GBM and suggests limited cell–cell interaction in the TME

Single‐cell RNA sequencing (scRNA‐seq) is a powerful analytical tool that provides a comprehensive and unbiased description of various cell types within a specific tissue. Although numerous studies have shown the effectiveness of this technique, it also possesses a notable drawback. This limitation arises from the necessity to break down the tissue before sequencing, resulting in the loss of spatial information. This information is essential for understanding the cellular interactions and tissue organization within the TME. Recently, researchers have overcome this limitation by integrating spatial transcriptomics with single‐cell RNA sequencing. The integration method described here applies not only to organizations with complex structures, but also has the potential for application in the entire field of biology.^53^

To better understand the distribution of various cell types in GBM tissues, we utilized the spatial transcriptomics (ST) method to analyze frozen sections of cancerous and adjacent tissues from four GBM patients within the GSE235672 dataset. We aimed to create an unbiased mapping of the transcripts expressed in these tissues. We initially utilized the Seurat package to integrate the ST sequencing data. Consequently, we evaluated the number of genes (nFeature_Spatial), mRNA molecules (nCount_Spatial), and the percentage of mitochondrial genes (percent.mt) in all cells of the ST data. The results demonstrated that most cells exhibited nFeature_Spatial values below 10,000, nCount_Spatial values below 50,000, and a percent.mt value below 5% (Figure S4A).

The results of the correlation calculation for sequencing depth reveal that there is a correlation coefficient of NA between the filtered data nCount_Spatial and percent.mt, and a correlation coefficient of 0.97 between nCount_Spatial and nFeature_Spatial (Figure S4B), suggesting that the ST data demonstrate high quality and are suitable for subsequent analysis. Figure S4C shows the distribution of nCount_Spatial on each organizational slice. The cell cycle of the sample was determined by utilizing the CellCycleScoring function (Figure S4D), followed by data standardization (Figure S4E).

**FIGURE 4:**
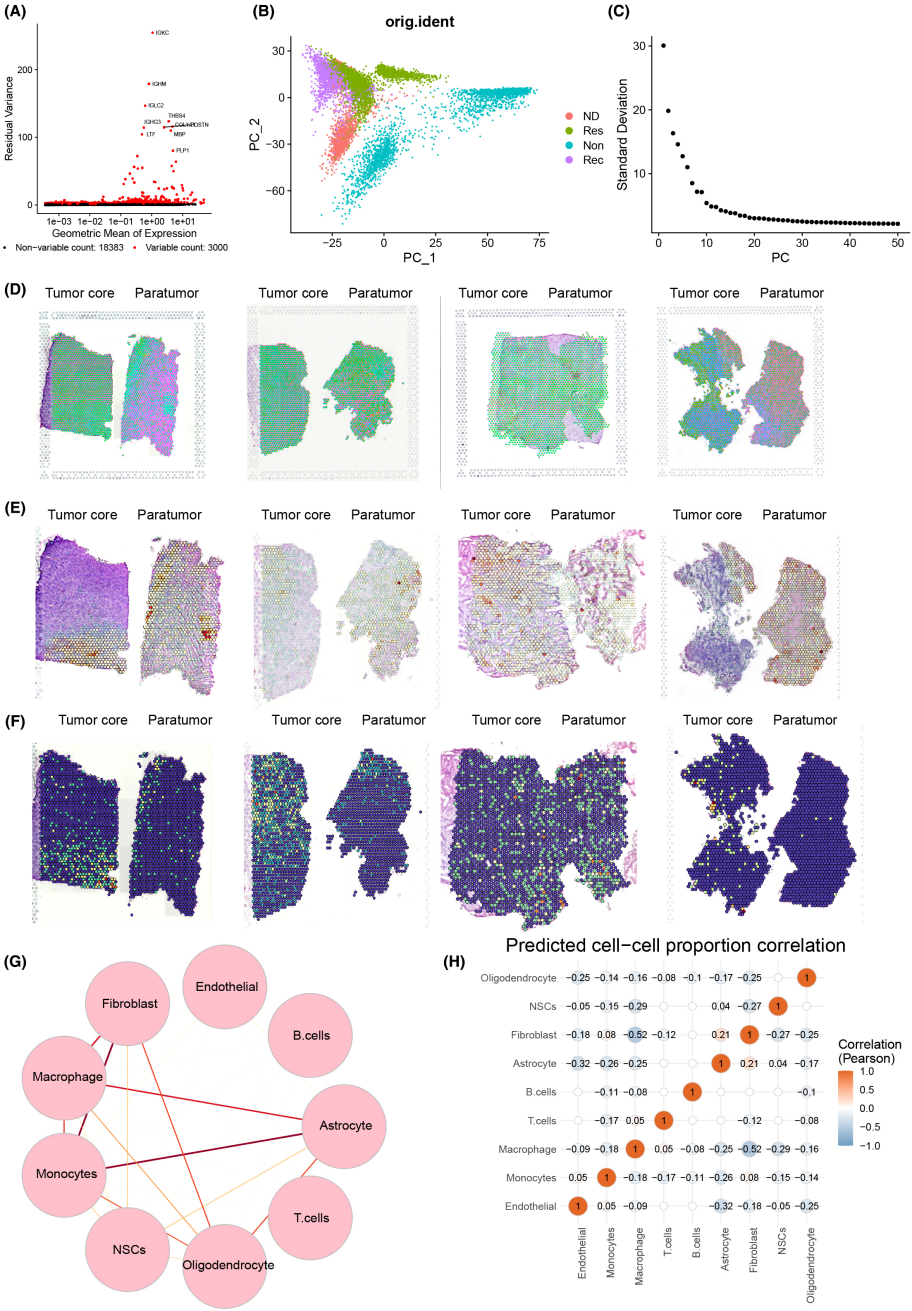
Analysis results of scRNA‐seq combined with ST. (A) Variance analysis to identify highly variable genes, with red indicating the top 3000 highly variable genes and black indicating low‐variable genes, labeling the top 10 genes in highly variable genes (*n* = 4). (B) PCA analysis of cell distribution in PC_1 and PC_2, with each point representing a cell (*n* = 4). (C) Distribution of standard deviation of PCs, with important PCs having larger standard deviations (*n* = 4). (D) Distribution of different cell types on ST data from PAAD tissue sections (*n* = 4), with pie charts representing the distribution of different cells in each spot. (E) Distribution of macrophages on ST data from PAAD tissue sections (*n* = 4), with increasing red color indicating a higher percentage of macrophages in that spot. (F) Expression of MS4A4A in different samples on ST data (*n* = 4). (G) Circular plot showing the interaction strengths between different cells on ST data, with thicker lines indicating stronger interaction (*n* = 4). (H) Heatmap showing cell correlations on ST data, with numerical values indicating correlation coefficients between cells (*n* = 4).

In further analysis of the ST data, we performed downstream analysis by filtering genes with high expression variance and selecting the top 3000 genes based on their ranking by variance (Figure 4A). Subsequently, we conducted PCA to lower the dimensionality of the selected highly variable genes and generated a plot of the PCA (Figure 4B). Additionally, we applied the ElbowPlot method to arrange the standard deviation of the PCs (Figure 4C). Figure S4F displays the gene expression heatmap associated with PCs 1–6. The results demonstrate that PCs 1 through 14 effectively capture the information in the selected highly variable genes and hold analytical value.

Next, we employ the TSNE algorithm to reduce nonlinear dimensionality on the initial 30 PCs. Subsequently, we choose a resolution of 0.4 for clustering analysis. We calculated the degree of overlap between genes identified from a specific cell type in a given tissue region and scRNA‐seq data to determine the enrichment of specific cell types in the designated tissue region. We then annotated the cells in the ST data (Figure 4D). The spatial distribution of macrophages and the expression of MS4A4A are visualized in Figure 4E,F, demonstrating the high expression of MS4A4A in the macrophage cluster.

We utilized the “SPOTlight” package in the R language to acquire spatial interaction data of cells. Circular plots of cell–cell interaction strength and a heatmap of cell correlation were generated (Figure 4G,H). The findings suggest a weak interaction between macrophages and other cells within the GBM TME. It implies that macrophages may exert independent effects in the TME rather than influencing tumor growth or treatment response through interaction with other cell types.

The results above suggest that MS4A4A‐positive macrophage clusters are more abundant in tumor tissue, spatially and in terms of expression levels. Furthermore, there is an increase in MS4A4A expression in the Non group compared to the Res group.

### 3.5 MS4A4A modulates TAM polarization and induces ferroptosis, influencing GBM development and immune suppression

To further validate the role of MS4A4A in TAMs in GBM, we initially confirmed the knockdown of MS4A4A in TAMs through RT‐qPCR and western blot techniques (Figure S5A). Subsequently, we chose the most effective sh‐MS4A4A‐1 for subsequent experiments.

**FIGURE 5:**
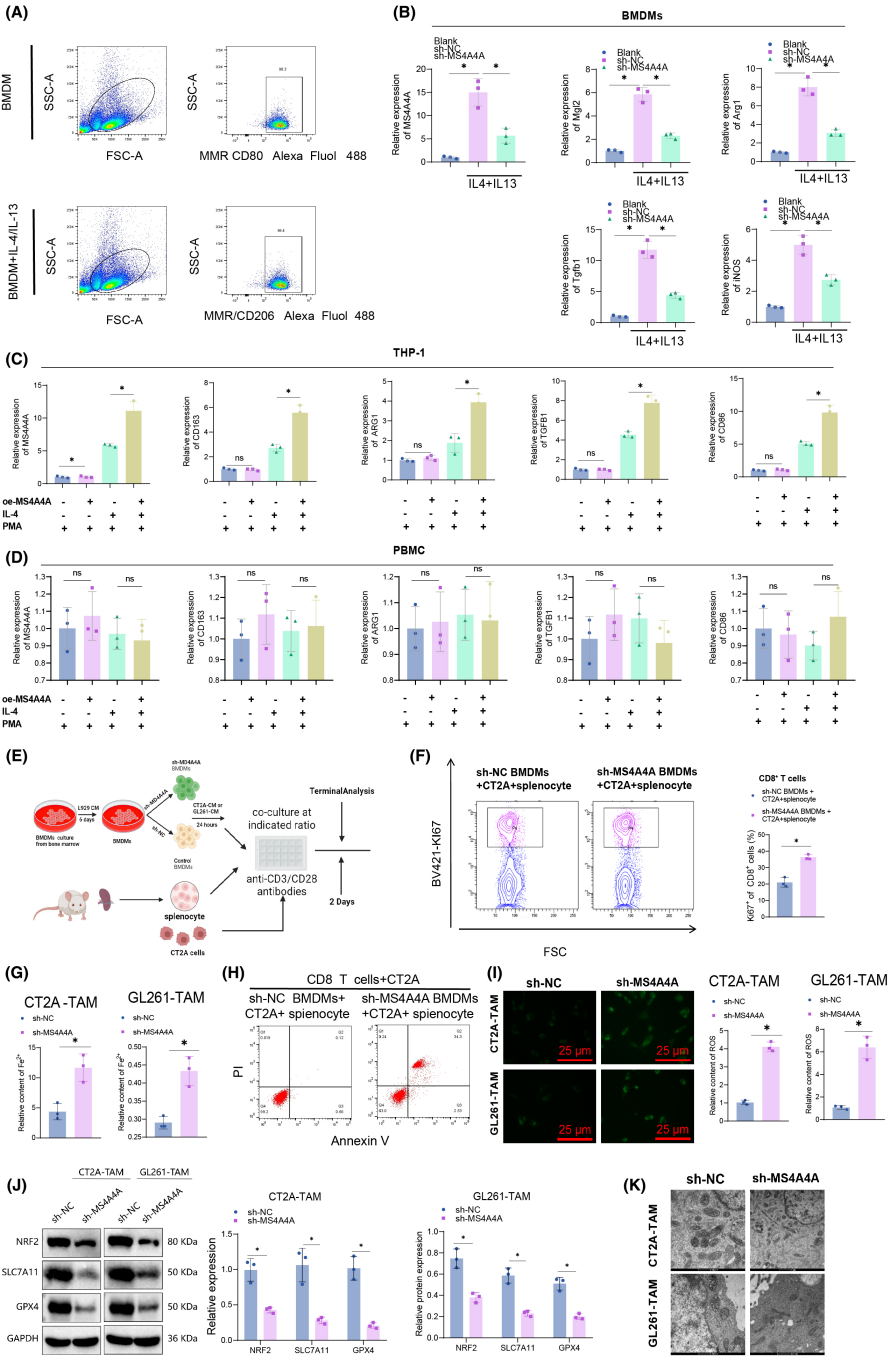
Inhibition of GBM cancer cell development by suppressing M2 macrophage infiltration through activation of TAM‐iron death pathway. (A) Flow cytometry was used to determine the proportion of M2 macrophages. (B) BMDMs were transfected with lentivirus for knockdown of MS4A4A or negative control plasmid, then induced into M2 phenotype using IL‐4 (20 ng/mL) and IL‐13 (20 ng/mL). qRT‐PCR was used to measure the interference efficiency of MS4A4A and expression of M2 markers (Mgl2, Arg1, and Tgfb1) and M1 marker iNOS. (C) THP‐1 human monocytic cell line was used to establish an overexpression cell line of MS4A4A, followed by differentiation into M0 macrophages using PMA (50 ng/mL). M0 macrophages were then polarized into M2 phenotype using IL‐4 (20 ng/mL) and qRT‐PCR was used to measure expression levels of MS4A4A and M2 markers (CD163, ARG1, and TGFB1) and M1 marker CD86. (D) PBMC cells were used to construct a cell line overexpressing MS4A4A. (E) Procedure for testing macrophage inhibition function: Mouse BMDMs and spleen cells were mixed with CT2A/GL261 cells, treated with MS4A4A antibody, and CD8^+^ T cells were sorted using flow cytometry and Ki‐67 expression was measured. (F) Analysis of Ki‐67 expression on designated CD8^+^ T cells using flow cytometry (*n* = 3). (G) Apoptosis of CT2A cells was detected using fluorescence‐activated cell sorting (FACS). (H) Iron content in tumor‐associated macrophages (TAMs) of sh‐NC and sh‐MS4A4A groups. (I) Reactive oxygen species (ROS) content in TAM cells of sh‐NC and sh‐MS4A4A groups. (J) Expression of iron death‐related proteins in TAM cells of sh‐NC and sh‐MS4A4A groups. (K) Electron microscopy analysis of TAM cells in sh‐NC and sh‐MS4A4A groups. *p < 0.05, and all cell experiments were repeated three times.

To examine the role of MS4A4A in regulating M2 polarization, the expression of MS4A4A in BMDMs was disrupted by transfecting them with lentivirus‐mediated MS4A4A knockdown plasmids. By conducting flow cytometry analysis to compare the proportions of M1 and M2 macrophages in BMDMs following treatment with either BMDM alone or IL4/IL13, our findings reveal a significant increase in the proportion of M2 macrophages in BMDMs after IL4/IL13 treatment (Figure 5A). The BMDMs were then polarized into an M2 phenotype by adding interleukin (IL)‐4/IL‐13. Compared to the control group sh‐NC, we observed a reduction in the expression of M2 markers (Mgl2, Arg1, and Tgfb1) in the sh‐MS4A4A group. Conversely, iNOS (inducible nitric oxide synthase) exhibited the highest specificity as a marker for M1 macrophages. Additionally, the results demonstrated an upregulation of iNOS in the MS4A4A overexpression group (Figure 5B).

Subsequently, lentivirus was used to create a stable overexpressing THP‐1 cell line of MS4A4A. The THP‐1 cells were then induced to differentiate into M0 macrophages by PMA and subsequently polarized into the M2 subtype using IL‐4. The results revealed an increase in M2 marker expression within the overexpressed MS4A4A group compared to the control group (Figure 5C). Inducible nitric oxide synthase (iNOS) is the most specific marker for M1 macrophages due to their production of nitric oxide as a crucial component of their antitumor effects.^54^ The results indicate a decrease in CD86 expression in the MS4A4A overexpressed group compared to the control group. The experimental results above indicate that MS4A4A is involved in macrophage polarization regulation. As a control, we overexpressed MS4A4A in PBMCs and treated them with IL‐4/IL‐13. However, due to the lack of potential for differentiation into macrophages in PBMCs, we did not observe any significant changes in the expression levels of macrophage markers posttreatment (Figure 5D).

To evaluate the influence of TAMs on the biological activities of GBM cells, we employed primary cells sourced from the mouse bone marrow in an in vitro experimental setting. We employed lentiviral transduction of MS4A4A knockdown plasmids to disrupt the expression of MS4A4A in BMDMs. Subsequently, we induced their differentiation into TAMs by supplementing IL‐4/IL‐13. Subsequently, we stimulated GBM cells with supernatant from TAMs and performed CCK8, EDU, Transwell, and scratch assays. The results demonstrate that TAMs markedly diminished the proliferative capacity of GBM cells in the sh‐MS4A4A group (Figure S5B).

To further confirm the involvement of TAMs in the proliferation of GBM cells, we conducted experiments using EDU staining. In comparison to the control group, TAMs in the sh‐MS4A4A group exhibited a reduction in the percentage of GBM cells positive for EDU staining, thereby providing further evidence of their inhibitory impact on cell proliferation (Figure S5C).

Additionally, we assessed the influence of TAMs on the invasion and migration of GBM cells using Transwell experiments. The experimental results demonstrated that the sh‐MS4A4A group experienced a decrease in the invasion and migration capabilities of GBM cells due to TAMs (Figure S5D). The results of the scratch experiment demonstrated that TAMs substantially decreased the migration speed of GBM cells in the sh‐MS4A4A group (Figure S5E,F).

Based on the experimental results presented earlier, it could be concluded that the expression of MS4A4A in TAMs enhances the proliferation, invasion, and migration of GBM cells. Thus, inhibiting the interference of MS4A4A could potentially serve as an effective approach to suppress the protumorigenic effects of TAMs on GBM cells. These findings present robust experimental evidence, which warrants further investigation into the role of MS4A4A in the TME and its potential as a therapeutic target.

To replicate the interaction between tumor cells and macrophages within the TME, we generated CM from mouse GBM cells and utilized it to culture BMDMs with varying levels of MS4A4A expression, prompting their transformation into TAMs (Figure S6A). The results revealed that when compared with the sh‐NC group with the addition of CT2A‐CM/GL261‐CM, the expression of MS4A4A and ARG1 mRNA in macrophages was upregulated by sh‐NC. However, when the expression of MS4A4A is disrupted, the regulation of CM on macrophage ARG1 mRNA expression is greatly reinstated (Figure S6B,C).

Furthermore, flow cytometry analysis (FACS) demonstrated that interference with MS4A4A expression decreased CD206 protein expression in TAMs. It suggests that the absence of MS4A4A reduced the proportion of M2‐like macrophages in TAMs (Figure S6D,F). Furthermore, we established J774A.1 and RAW264.7 macrophage cell lines overexpressing MS4A4A for conducting the corresponding experiments. Compared to the sh‐NC group with CM from CT2A and GL261, the macrophages that overexpressed MS4A4A exhibited an increase in the expression of ARG1 mRNA upon adding the same CM. This finding suggests that the overexpression of MS4A4A could augment M2 polarization (Figure S6E,G). The experiments above have substantiated the significance of MS4A4A in governing the behavior of macrophages.

Subsequently, we induced differential levels of MS4A4A expression in BMDMs by treating them with CT2A‐CM/GL261‐CM and collected the supernatant from TAM cultures. Our results demonstrate that the expression of anti‐inflammatory cytokines IL‐10 and TGF‐β1 in the supernatant of TAM cultures was decreased in the MS4A4A interference group compared to the control group (Figure S6H,I). Conversely, overexpression of MS4A4A led to an increase in the levels of IL‐10 and TGF‐β1 in the TAM culture supernatant (Figure S6J,K). These findings suggest that MS4A4A^+^ macrophages possess enhanced immunosuppressive capabilities.

Given that the primary pathogenic function of TAM is to impede the antitumor immune response, we subsequently assessed the inhibitory impact of TAM's MS4A4A expression on the ability of T cells to proliferate (Figure 5E). Compared to the sh‐NC group, the suppression of MS4A4A expression substantially increased the proliferative capacity of CD8^+^ T cells cocultured with TAM, suggesting that TAM reduces the inhibitory effect on T cells (Figure 5F). Coculturing CD8 T cells with TAMs transfected with sh‐NC or sh‐MS4A4A and CT2A cells, a propidium iodide staining assay was performed to detect the apoptosis rate of tumor cells. The results indicate a significant increase in the proportion of apoptotic tumor cells in the sh‐MS4A4A group, suggesting a remarkable alleviation of M2 macrophage‐mediated suppression on T cells upon MS4A4A depletion (Figure 5G).

In conclusion, these findings suggest that MS4A4 plays a role in promoting M2‐TAM polarization, which leads to a more immunosuppressive TME.

How MS4A4A modifies the TAM phenotype is a captivating scientific inquiry that merits further investigation. Ferroptosis constitutes the primary programmed cell death pathway in GBM. Iron cell death is associated with a negative prognosis and immune suppression in GBM.^55^

It is noteworthy that TAMs play a crucial role in immune suppression associated with iron death. We initially quantified the iron levels in TAM to confirm the association between MS4A4A and iron‐mediated cell death in TAM cells from the sh‐NC and sh‐MS4A4A groups. In our in vitro experiments, we stimulated BMDM cells with GBM cell supernatant to evaluate the impact of varying levels of MS4A4A expression on TAMs' iron content. The results suggest that the iron content is higher in the sh‐MS4A4A group compared to the sh‐NC group (Figure 5H).

Furthermore, we quantified the ROS levels in TAM. The findings indicate that the levels of ROS were higher in the sh‐MS4A4A group compared to the sh‐NC group (Figure 5I). The Western blot analysis also confirmed the downregulation of iron death‐related proteins, including GPX4, NRF2, and SLC7A11, in the sh‐MS4A4A group. This finding suggests that the knockdown of MS4A4A expression in TAM increases iron death (Figure 5J). The electron microscopy image reveals that the mitochondria in the sh‐MS4A4A group treated with TAM exhibit reduced size and volume, increased electron density, and expanded ridges. These findings are consistent with the features of iron death (Figure 5K).

The collective findings from the experiments earlier suggest that the blockade of MS4A4A in the GBM microenvironment triggers the iron death signaling pathway in TAMs, leading to a decrease in the presence of M2‐type macrophages and promoting a transition from M2 to M1 macrophages. This transformation inhibits the proliferation and invasion of GBM cells. Therefore, MS4A4A plays a crucial regulatory role in modulating TAM phenotypes and influencing GBM development, providing a robust theoretical foundation for developing novel immunotherapeutic strategies targeting GBM.

### 3.6 MS4A4A regulates macrophage polarization and T‐cell functionality in GBM, influencing tumor growth and immune response dynamics

Macrophages are highly prevalent immune cells infiltrating GBM and are closely linked to tumor progression and treatment effectiveness.^56^

**FIGURE 6:**
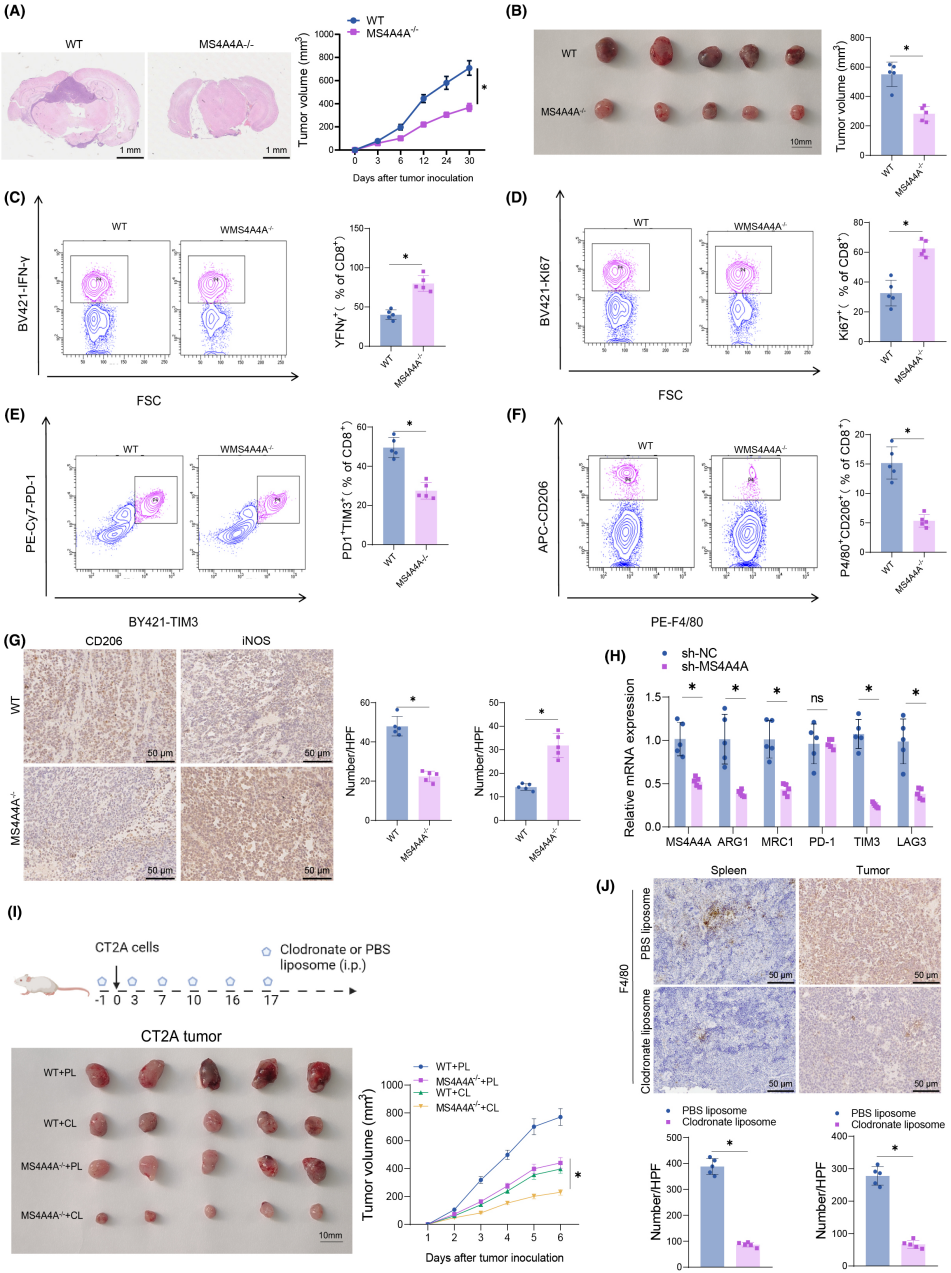
Inhibition of macrophage activation by MS4A4A delays the progression of GBM. (A, B) Tumor growth after intracranial/subcutaneous injection of CT2A cells in WT and MS4A4A^−/−^ mice (*n* = 5 per group). (C–F) FACS analysis of infiltrating CD8^+^ T cells and TAM‐specific markers in CT26 tumor‐bearing mice. (G) Immunohistochemical staining of CD206^+^ and iNOS^+^ macrophage infiltration in subcutaneous CT2A tumors using specific antibodies CD206 and iNOS. The number of CD206‐ and iNOS‐positive cells was calculated for each high‐power field (HPF) in the subcutaneous tumor sections, and five random HPFs were analyzed. (H) Relative expression levels of related genes measured by qRT‐PCR. (I) WT and MS4A4A^−/−^ mice were subcutaneously injected with CT2A cells on day 0 and treated with an intraperitoneal injection of PBS liposomes (PL) or clodronate liposomes (CL) on the day before cell implantation and days 3, 7, 10, 16, and 17 after cell implantation to deplete macrophages (*n* = 5). (J) Immunohistochemical staining of F4/80‐positive macrophages in the spleen and tumor of MS4A4A^−/−^ mice with subcutaneous CT2A tumors. The number of F4/80 positive cells was calculated for each HPF in the subcutaneous tumor sections, and five random HPFs were analyzed. *p < 0.05 compared to the control group, *n* = 5.

In our study, we initially generated MS4A4A knockout mice and verified the effectiveness of the knockout using the methodology demonstrated in Figure S8F. Subsequently, we injected CT2A and GL261 glioma cells into MS4A4A knockout mice to further explore the influence of MS4A4A on tumor growth. The results demonstrated that inhibiting MS4A4A could considerably impede tumor growth, as evidenced by the data presented in Figure 6A,B and Figure S7A,B.

Further analysis of the composition of immune cells (Figure S8A–E) revealed that MS4A4A knockout not only improved the antitumor immune response in mice but also led to a marked decrease in macrophage abundance and an increase in CD8^+^ T‐cell abundance within the TME. We further investigated this change's impact on T cells' functionality, as depicted in Figure 6C,D and Figure S7C,D.

The results suggest that the knockout of MS4A4A enhances the activity of tumor‐infiltrating CD8^+^ T cells, as determined by evaluating their activity through the expression levels of IFN‐γ and Ki67. Furthermore, the exhaustion markers PD‐1 and TIM3 on T cells and the infiltration levels of M2 TAMs showed a reduction (Figure 6E,F and Figure S7E,F). Immunohistochemical staining revealed a notable reduction in the count of CD206‐positive cells and an elevation in the count of iNOS‐positive cells in the CT2A/GL261 subcutaneous transplant tumors of MS4A4A^−/−^ mice, as compared to the WT group (Figure 6G; Figure S7G).

The results of the qRT‐PCR experiment revealed a decrease in the relative expression levels of M2 macrophage marker genes MCR1 and ARG1 in the tumor tissue of MS4A4A^−/−^ mice compared to the WT group. Furthermore, we investigated the immune checkpoint markers PD‐1, TIM3, and LAG3 on T cells. The results revealed a reduction in TIM3 expression (Figure 6H; Figure S7H). These findings imply that MS4A4A is linked to the immune evasion mechanisms within the TME.

To verify if the observed results are reliant on macrophages, we employed clodronate liposomes to deplete macrophages in vivo (Figure 6I,J; Figure S7I,J). Significantly, the growth inhibition of GBM by MS4A4A is markedly reduced when macrophages are removed.

Based on the experimental findings, we could conclude that in a physiological environment, the surface molecule MS4A4A on macrophages influences tumor growth and progression by regulating macrophage polarization and function. Specifically, inhibiting MS4A4A could boost the antitumor immune response, alleviate T‐cell exhaustion, and reduce the infiltration of M2‐type TAMs. Moreover, the tumor‐suppressive effect is contingent upon the presence of macrophages.

### 3.7 Targeting MS4A4A enhances the efficacy of PD‐1 immunotherapy in GBM by modulating the tumor immune microenvironment

While PD‐1/programmed death‐ligand 1 (PD‐L1) therapy primarily targets CD8^+^ T cells, it is important to note that these cells are tightly regulated by TAMs within the TME. Thus, targeting TAMs in treatment could serve as an additional potential approach for immunotherapy.^56^

**FIGURE 7:**
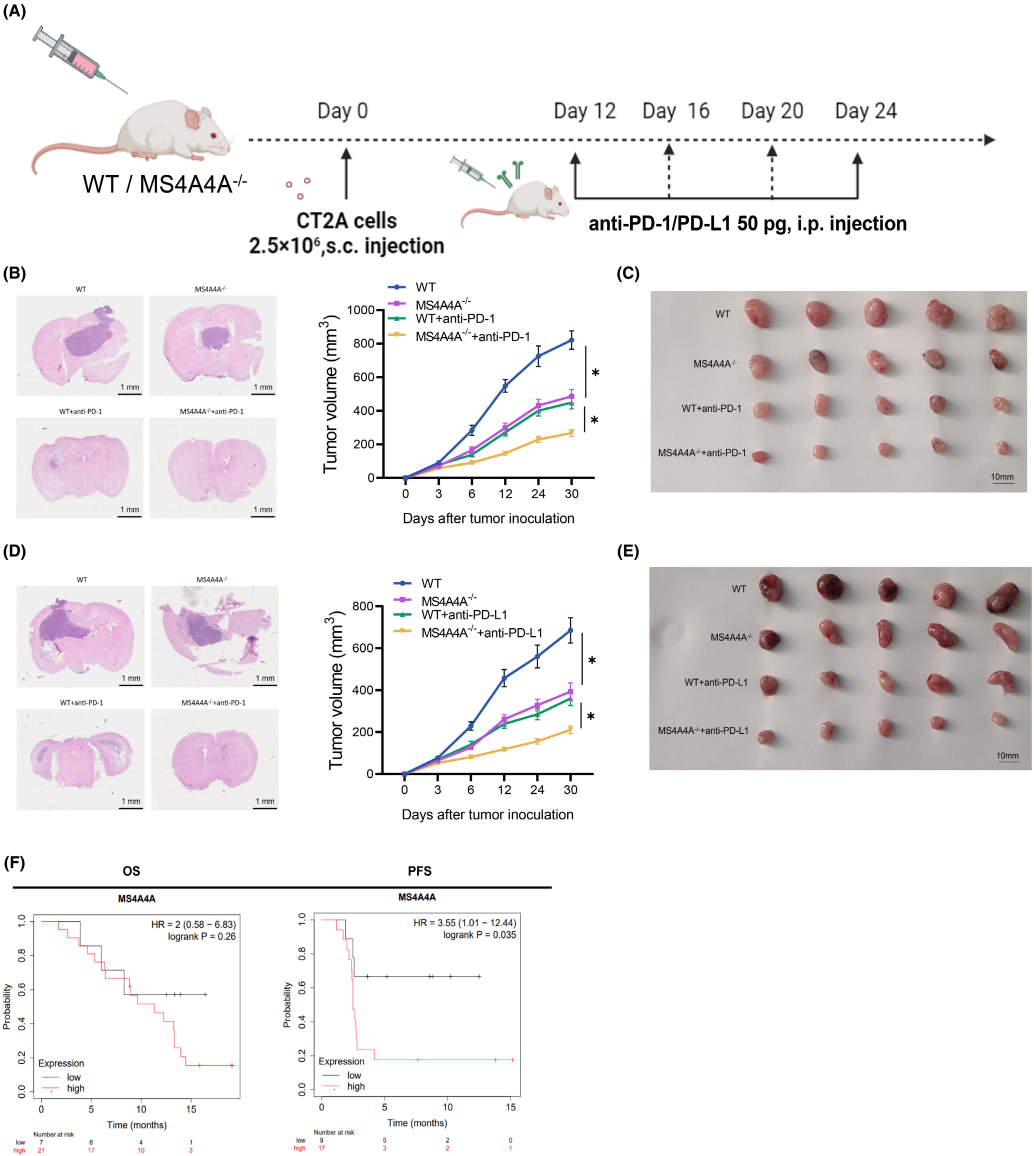
Targeting MS4A4A enhances the efficacy of anti‐PD‐1 immunotherapy. (A) Illustration of the animal model. (B–E) Tumor growth in each group of mice (*n* = 5 per group). (F) Kaplan–Meier plot showing the association between MS4A4A gene expression levels and overall survival or progression‐free survival of GBM patients. *p < 0.05 compared to the control group.

To examine the role of MS4A4A in regulating macrophage polarization and its impact on tumor growth in vivo, this study utilized a mouse model with MS4A4A knockout (MS4A4A^−/−^) and implanted CT2A cells to mimic GBM (Figure 7A). The findings revealed that the inhibition of MS4A4A, in combination with PD‐1 antibody treatment, led to a delay in tumor growth, demonstrating notable disparities compared to PD‐1 monotherapy (Figure 7B–E).

CD3 and CD8 are markers of T cells, with CD8 specifically identifying cytotoxic T cells (CTLs). Elevating their levels typically signifies immune activation and a heightened antitumor response. Immunohistochemical experiments demonstrated that targeting MS4A4A, in combination with PD‐1 antibody treatment, substantially enhanced the infiltration of CD3^+^ and CD8^+^ T cells in tumor‐bearing mice (Figure S9).

In our analysis using the Kaplan–Meier plotter online database (https://kmplot.com/analysis/), we found that patients with high MS4A4A expression in GBM usually exhibit decreased overall survival and progression‐free survival rates (Figure 7F).

These results demonstrate that MS4A4A acts as a crucial regulatory factor in suppressing immune responses and diminishing the effectiveness of anti‐PD‐1 immunotherapy. Targeting MS4A4A in conjunction with PD‐1 antibody treatment can potentially delay tumor growth and alter the tumor's immune microenvironment.

## 4 DISCUSSION

By employing single‐cell and spatial transcriptomic sequencing, we have uncovered that inhibiting MS4A4A can facilitate the transition of M2 macrophages toward M1 macrophages through ferroptosis. This process leads to the reshaping of the tumor immune microenvironment and enhances the effectiveness of anti‐PD‐1 immunotherapy for GBM. In contrast to existing immunotherapies for GBM, conventional single‐treatment approaches often yield limited results. The combined strategy proposed in this study, targeting M2 macrophages alongside PD‐1 immunotherapy, holds promise for improving the effectiveness of immunotherapy.^3^, ^57^, ^58^

**FIGURE 8:**
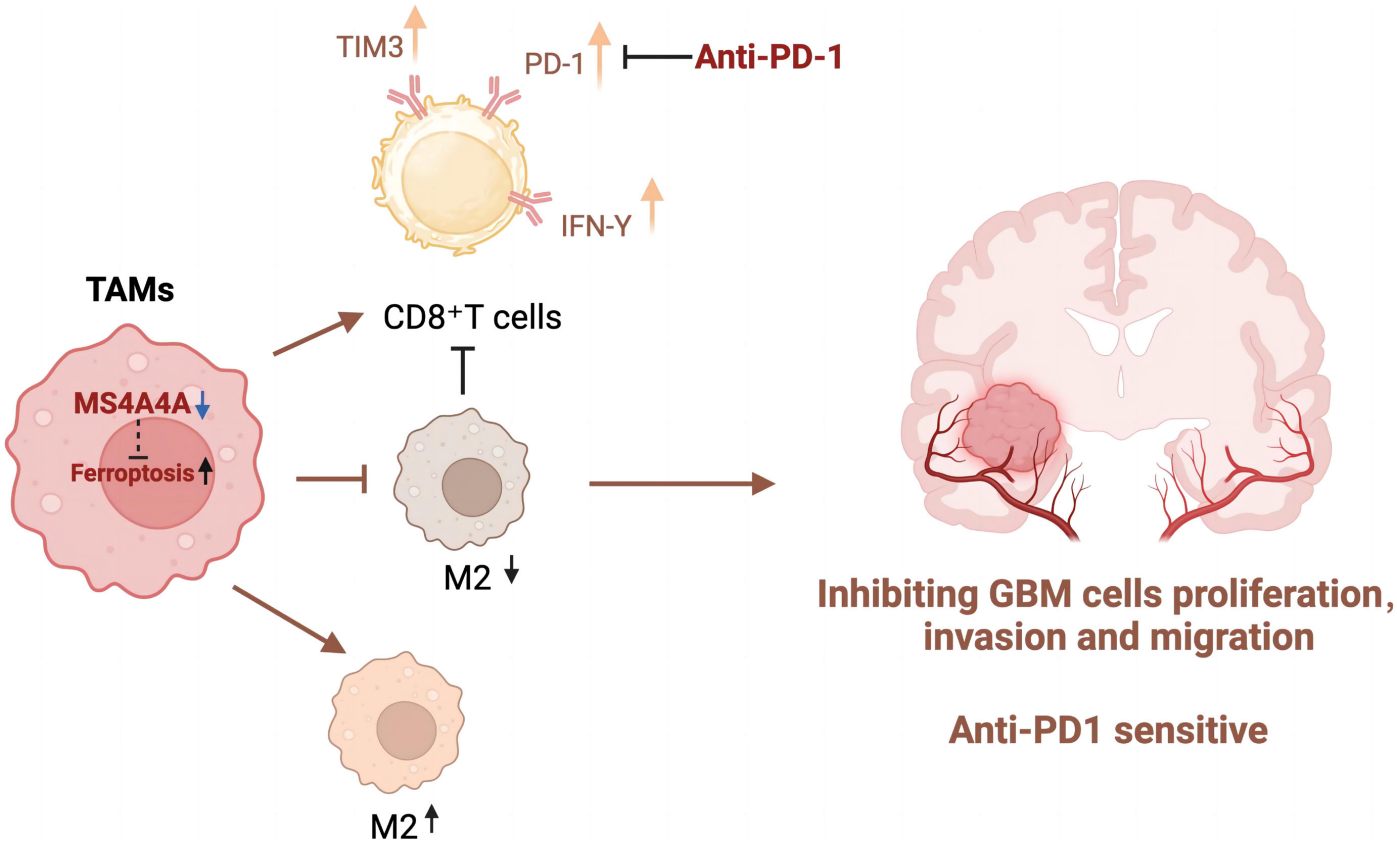
Molecular mechanism diagram depicting the promotion of M2 to M1 macrophage conversion and reshaping of the tumor immune microenvironment by suppressing MS4A4A through iron‐induced apoptosis.

Single‐cell sequencing has revealed that M2 macrophages in glioma tissue highly express MS4A4A, and its expression is positively correlated with the expression of immune‐suppressive genes.^59^ This finding aligns with previous studies on GBM immunotherapy, suggesting M2 macrophages impact immune evasion in GBM.^60^ This study further investigates the potential mechanisms by which MS4A4A regulates the polarization state of M2 macrophages and the expression of immune‐suppressive‐related genes. These findings provide a new theoretical basis for immunotherapy targeting GBM (Figure 8). In vitro cell experiments have confirmed that inhibiting MS4A4A leads to the transition of M2 macrophages to the M1 phenotype. Further investigations suggest that the ferroptosis pathway may be involved in this transition process. This discovery presents a novel direction for optimizing immunotherapy strategies for glioma. Nonetheless, a more thorough investigation is still needed to elucidate the specific mechanisms and regulatory signals of the ferroptosis pathway in the transition of M2 to M1 macrophages.

Above all, we employed single‐cell and spatial transcriptomic sequencing techniques to delve into the immunoregulatory mechanisms of M2‐type macrophages in gliomas.^61^ The discovery of the significance of MS4A4A in the immune microenvironment of GBM, along with its association with M2 macrophage polarization and immune suppression, has broadened our comprehension of the immune evasion mechanisms in GBM. This study uncovered a potential mechanism through which inhibition of MS4A4A promotes the conversion of M2‐like macrophages into M1‐like macrophages. It provides novel insights and a theoretical foundation for refining immunotherapy strategies for GBM. This study proposes a strategy to inhibit MS4A4A to enhance the immune microenvironment in gliomas and improve the efficacy of anti‐PD‐1 immunotherapy, offering new hope for the clinical translation of immune therapy for gliomas. This approach aims to enhance the efficiency and tolerance of immunotherapy, providing glioma patients with more effective treatment options.

This study presents preliminary evidence for MS4A4A as a potential target for immune therapy in gliomas, but its clinical translational prospects require further research and clinical validation. While macrophages decrease progressively in different stages of glioma development, the high expression of MS4A4A is associated with adverse survival outcomes in glioma patients. Additionally, the significant upregulation of MS4A4A gene expression in M2 macrophages of various tumors warrants verification in clinical populations, and the lack of relevant validation results stands as a limitation of this study. The research underscores MS4A4A as a potential target for immune therapy in gliomas. Aside from MS4A4A, there are other promising immune therapy targets warranting further investigation, such as immune checkpoints and immune regulatory factors. Targeting multiple immune regulatory factors in combination could significantly enhance the efficacy of immune therapy. Furthermore, glioma tissues harbor various cellular and chemical factors that inhibit immune responses, thus exploring methods to inhibit these factors is also worth further research. While this study conducted in vivo validation using a mouse glioma model, differences exist between animal models and human gliomas. Despite offering preliminary evidence, caution must be exercised in evaluating the clinical translational prospects of mouse models. Future focus should prioritize preclinical models and in vitro studies to gain a better understanding of the therapeutic potential of inhibiting MS4A4A in human gliomas. Crucially, the next step involves conducting clinical trials to validate the actual efficacy and safety of inhibiting MS4A4A in immune therapy for gliomas. Moreover, factors such as the complexity of immune therapy and individual variances must be thoroughly considered in clinical application. In conclusion, this study opens up new avenues for exploring novel targets in immune therapy for gliomas, yet further in‐depth investigation is required for its clinical implementation.

The next area of research should prioritize conducting clinical trials to confirm the therapeutic efficacy and safety of inhibiting MS4A4A for glioma immunotherapy. Clinical trials could further assess the therapeutic effectiveness of this strategy, thereby providing more reliable data to support its clinical application.^62^ Besides MS4A4A, additional immunotherapy targets exist within glioma tissue.^63^ Future studies could investigate combination strategies that target multiple immune regulatory factors to enhance the overall effectiveness of immunotherapy for GBM. The immune status and gene expression levels could vary substantially among patients with glioma. As a result, personalized treatment strategies may represent the future direction of immunotherapy. Treatment efficacy could be improved, and the risk of adverse reactions reduced through therapeutic strategies based on individual genetic information.

In summary, this study offers a novel avenue for investigating potential targets for glioma immunotherapy. Although this study confirms the involvement of the iron death pathway in the regulation of M2 to M1 macrophage transformation by MS4A4A, the specific regulatory pathways and mechanisms involved require further exploration. It is worth noting that we validated the strategy of improving the anti‐PD‐1 immunotherapy effect on GBM through the use of MS4A4A^−/−^ mice combined with ICB. The validation of this approach through specific PD‐1 blockade using small molecule inhibitors may lead to better clinical translational outcomes, which will also be the focus of our future research. Nevertheless, additional extensive research and validation are necessary before its application in clinical settings. As immunotherapy techniques continue to advance, it is anticipated that more innovative treatment strategies will be implemented for glioma treatment, leading to improved clinical outcomes for patients.

## AUTHORS CONTRIBUTIONS

GCS, XGC, and YLW wrote the article and conceived and designed the experiments; SYL, CYL, and YJ analyzed the data; DSC, NL, and XL collected and provided the sample for this study. All authors have read and approved the final submitted manuscript.

## FUNDING INFORMATION

This study was supported by the Liaoning Youth Science Project.

## CONFLICT OF INTEREST STATEMENT

The authors declare that they have no competing interests.

## Supporting information


Figure S1.

Figure S2.

Figure S3.

Figure S4.

Figure S5.

Figure S6.

Figure S7.

Figure S8.

Figure S9.

Tables S1–S4.

## Data Availability

The data that support the findings of this study are available on request from the corresponding author.
