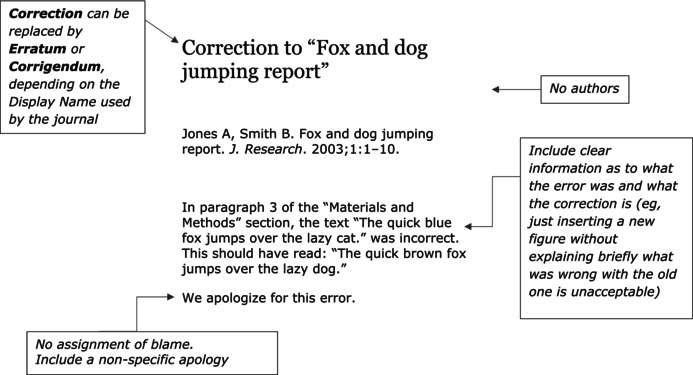# Correction to Targeting MS4A4A: A novel pathway to improve immunotherapy responses in glioblastoma

**DOI:** 10.1111/cns.70027

**Published:** 2024-09-03

Shao G, Cui X, Wang Y, et al. Targeting MS4A4A: a novel pathway to improve immunotherapy responses in glioblastoma. *CNS Neurosci Ther*. 2024;30:e14791. doi:10.1111/cns.14791

The fund, Liaoning Youth Science Project, has been closed and is no longer in use.

We apologize for this error.

Example