# Supplementary figures and images for: Targeting MS4A4A: A novel pathway to improve immunotherapy responses in glioblastoma
Source: CNS Neurosci Ther. 2024 Jul 12;30(7):e14791. doi: 10.1111/cns.14791 (PMC11245405; doi:10.1111/cns.14791)

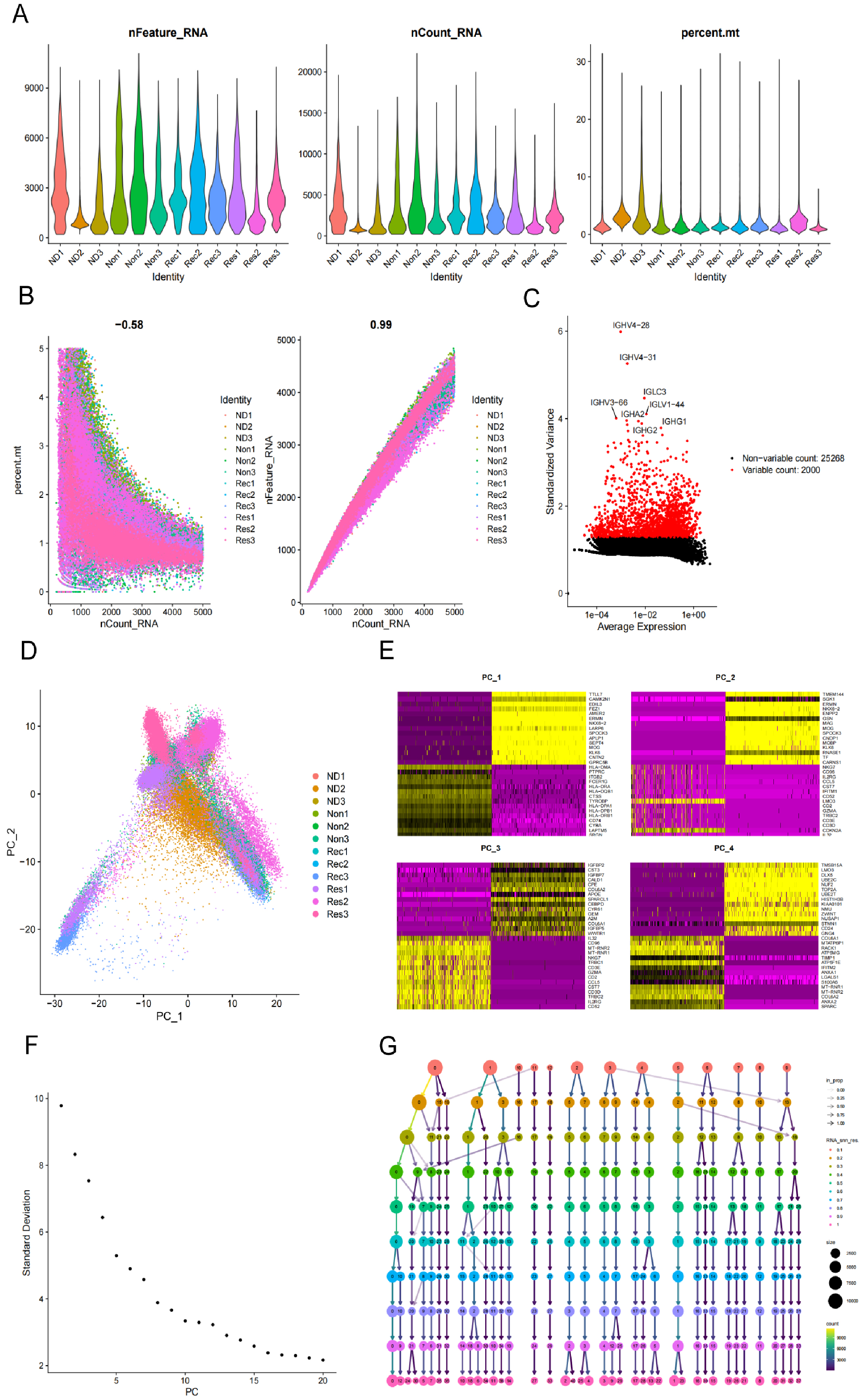

Supplement: Supplementary file 1 — Figure S1. [file CNS-30-e14791-s001.jpg]

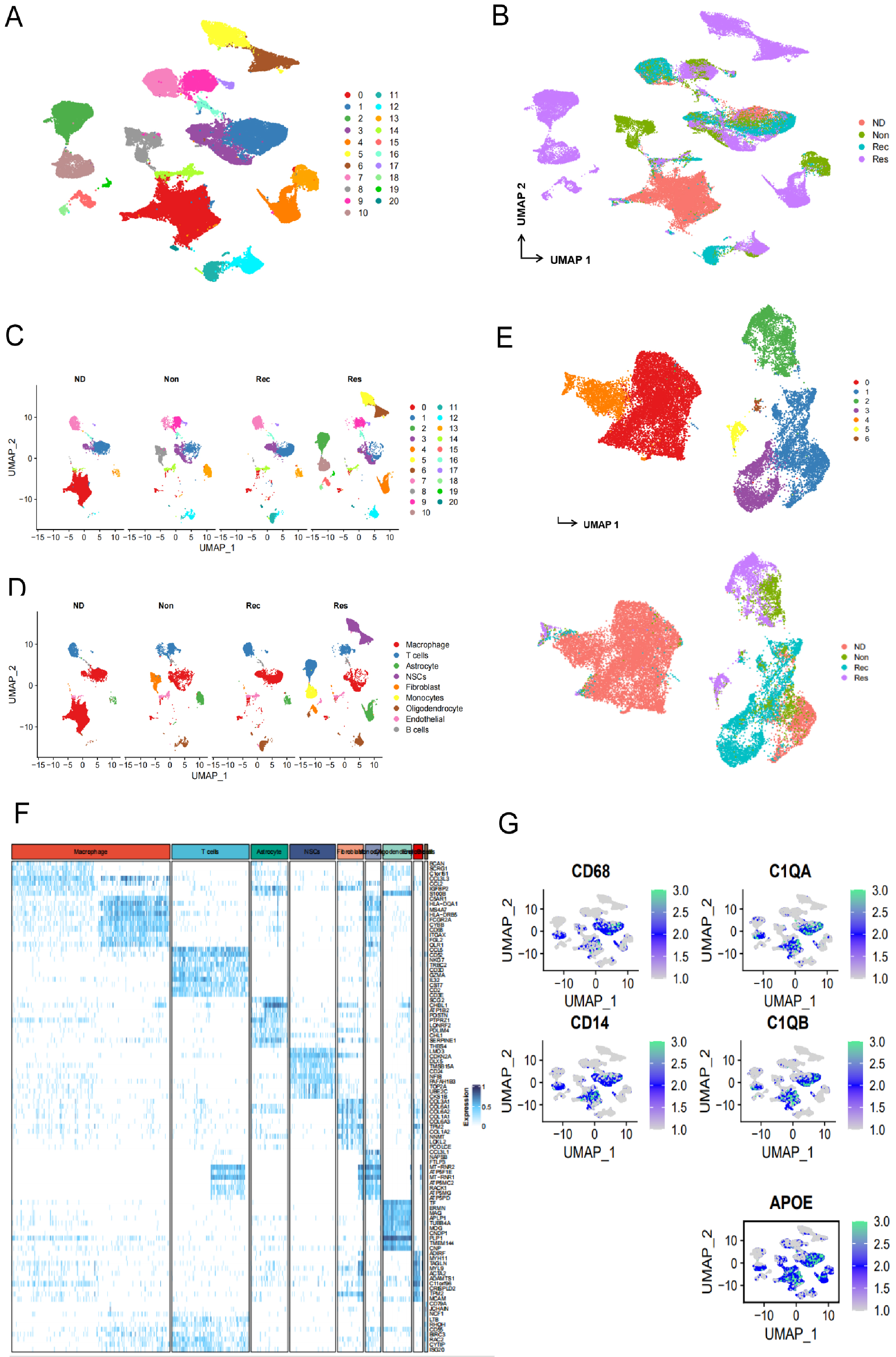

Supplement: Supplementary file 2 — Figure S2. [file CNS-30-e14791-s008.jpg]

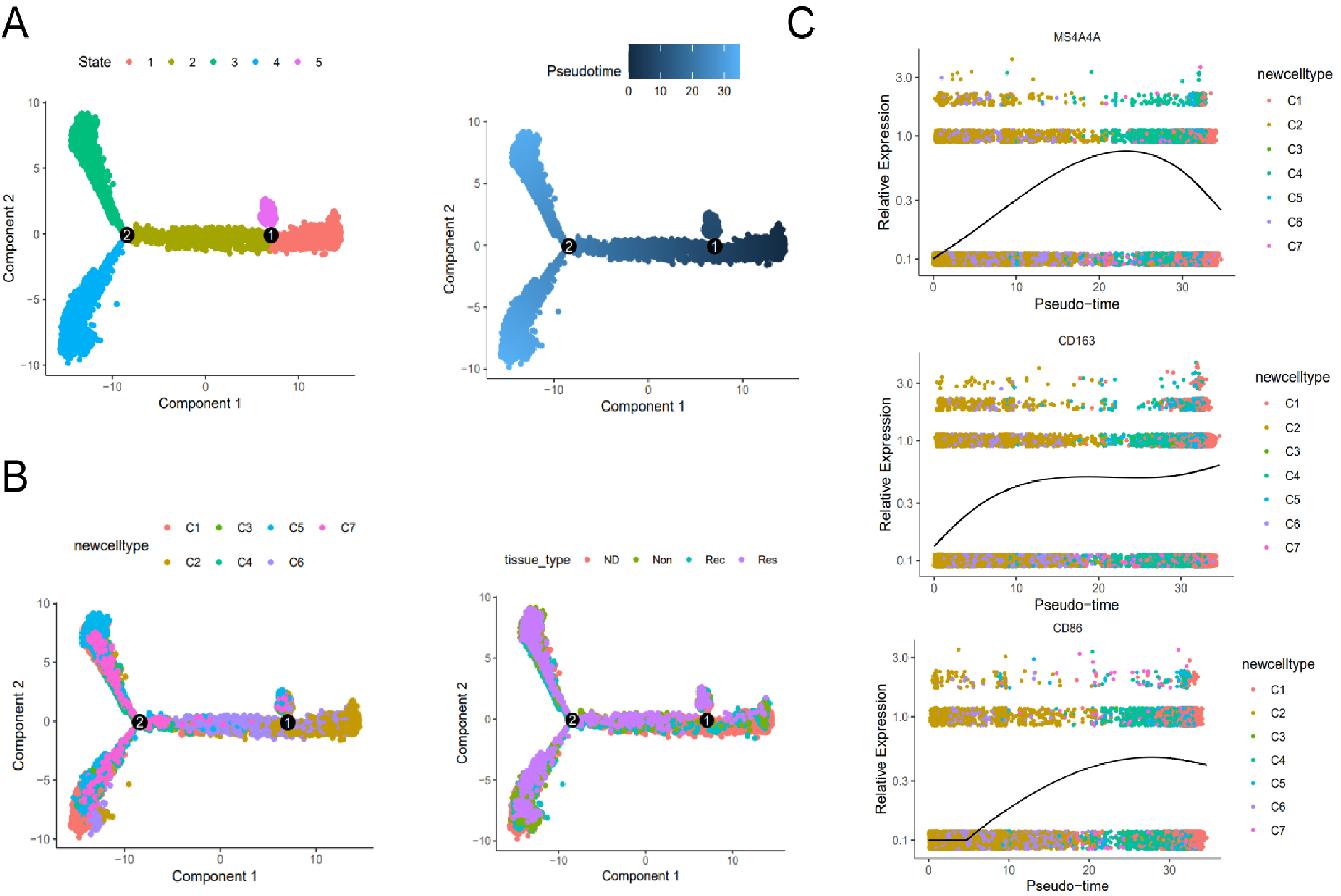

Supplement: Supplementary file 3 — Figure S3. [file CNS-30-e14791-s010.jpg]

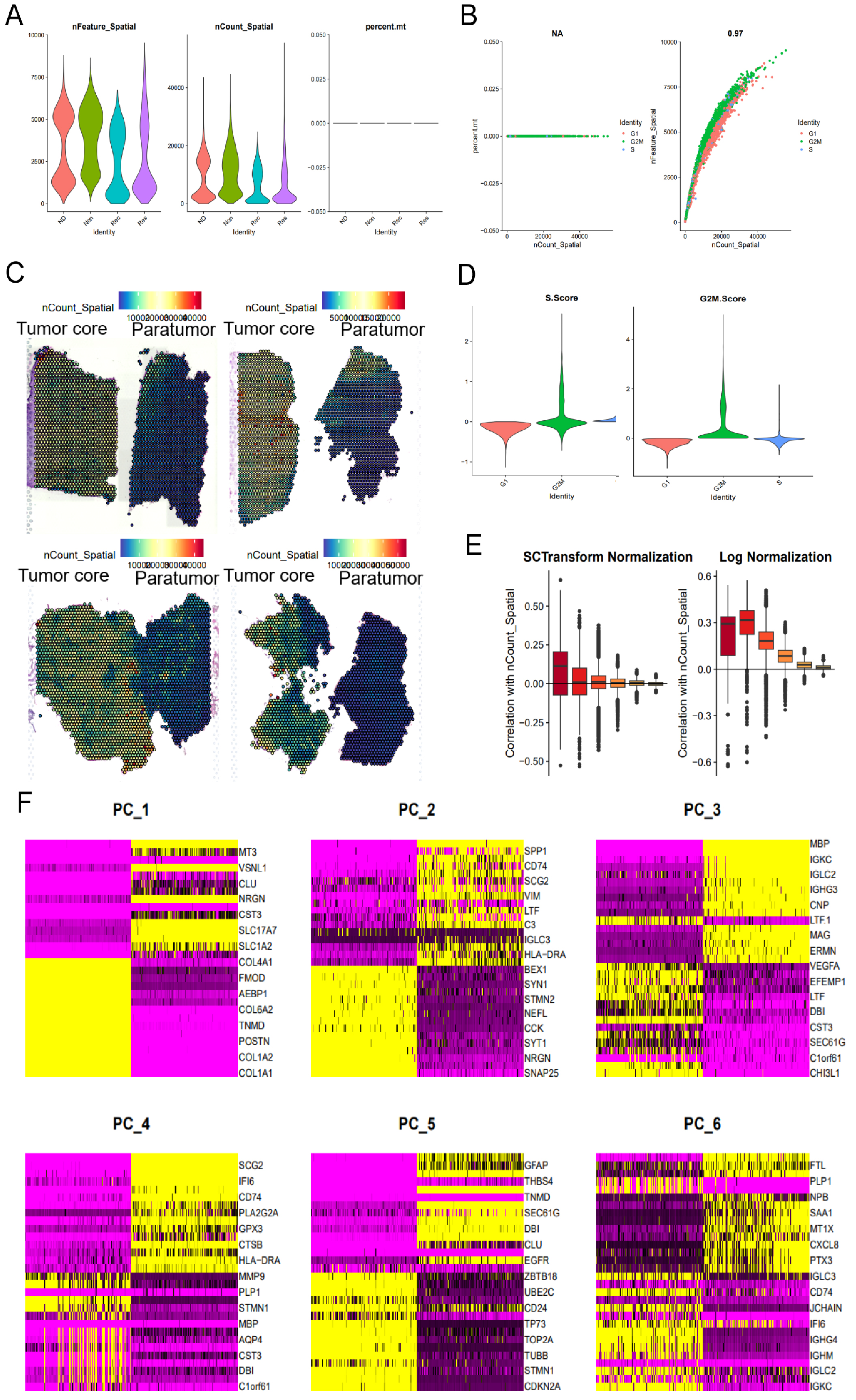

Supplement: Supplementary file 4 — Figure S4. [file CNS-30-e14791-s007.jpg]

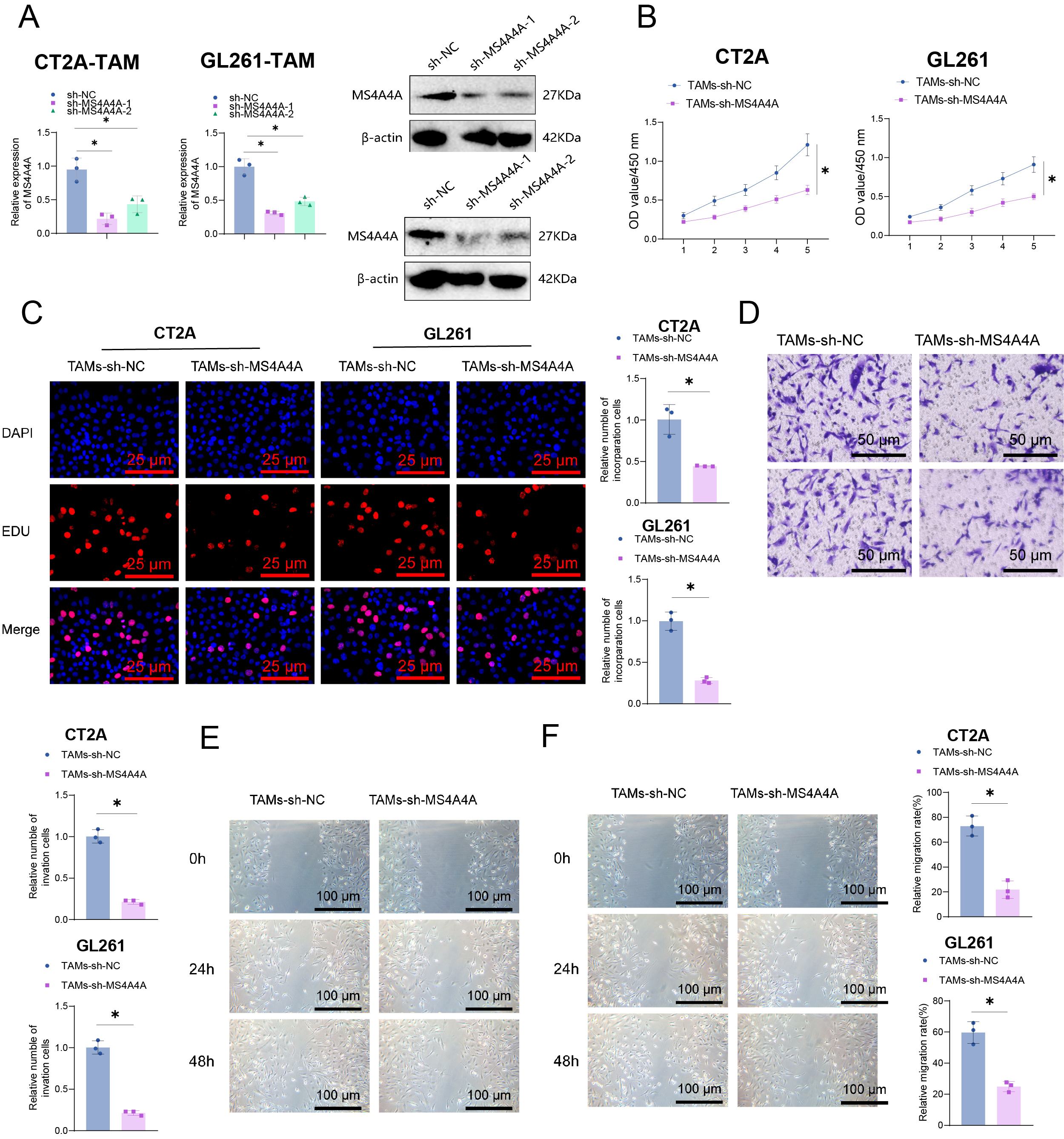

Supplement: Supplementary file 5 — Figure S5. [file CNS-30-e14791-s009.jpg]

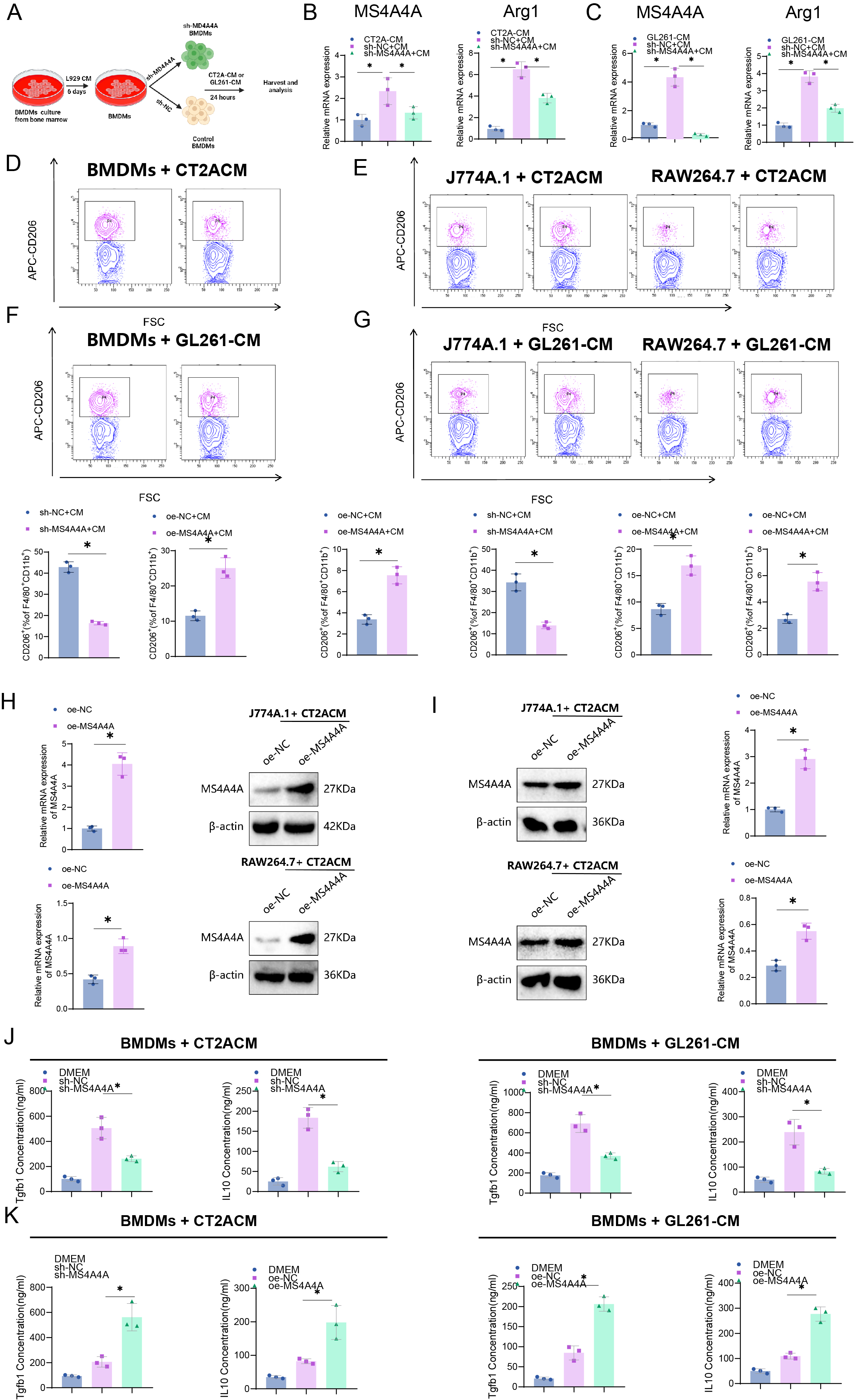

Supplement: Supplementary file 6 — Figure S6. [file CNS-30-e14791-s002.jpg]

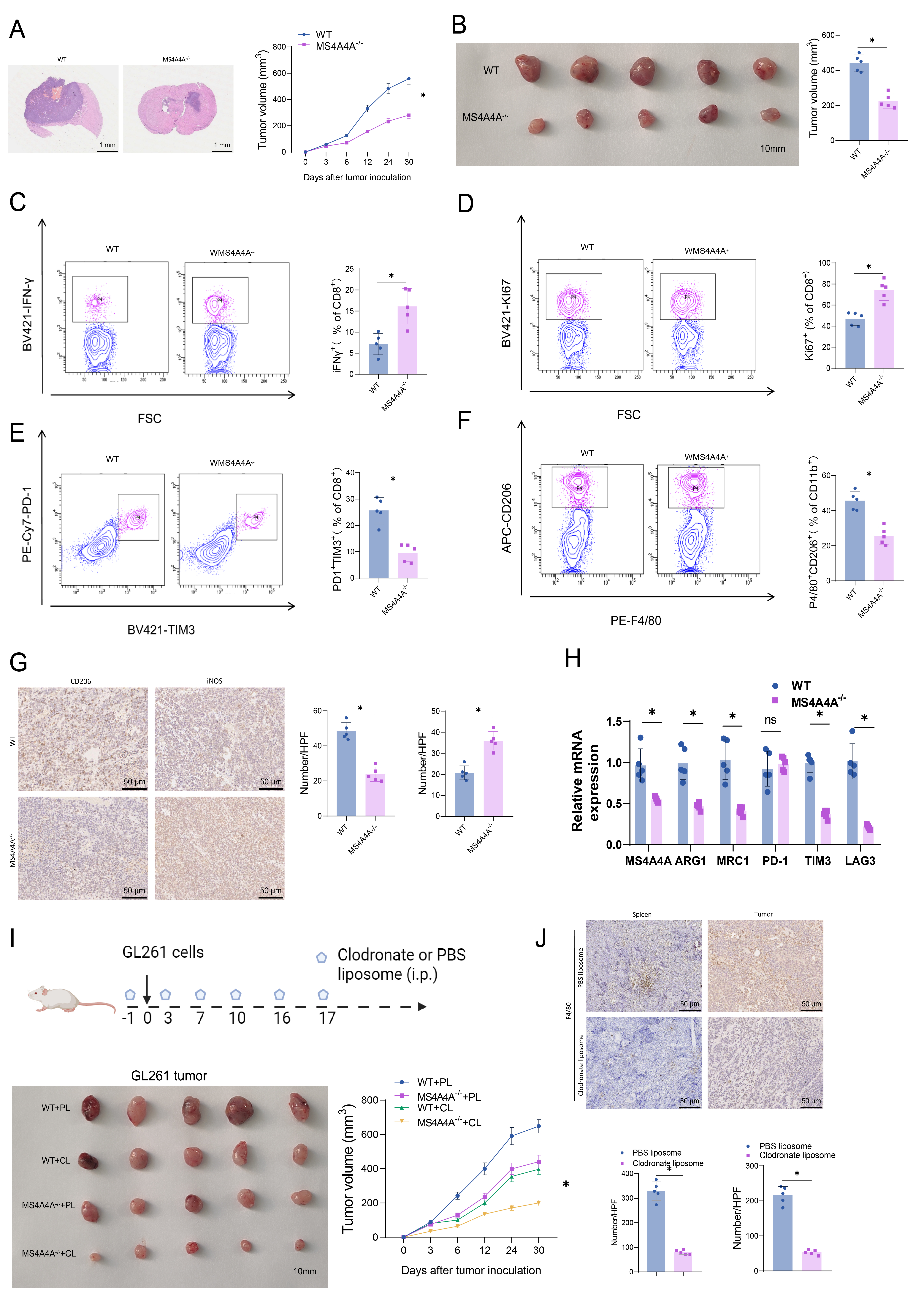

Supplement: Supplementary file 7 — Figure S7. [file CNS-30-e14791-s005.jpg]

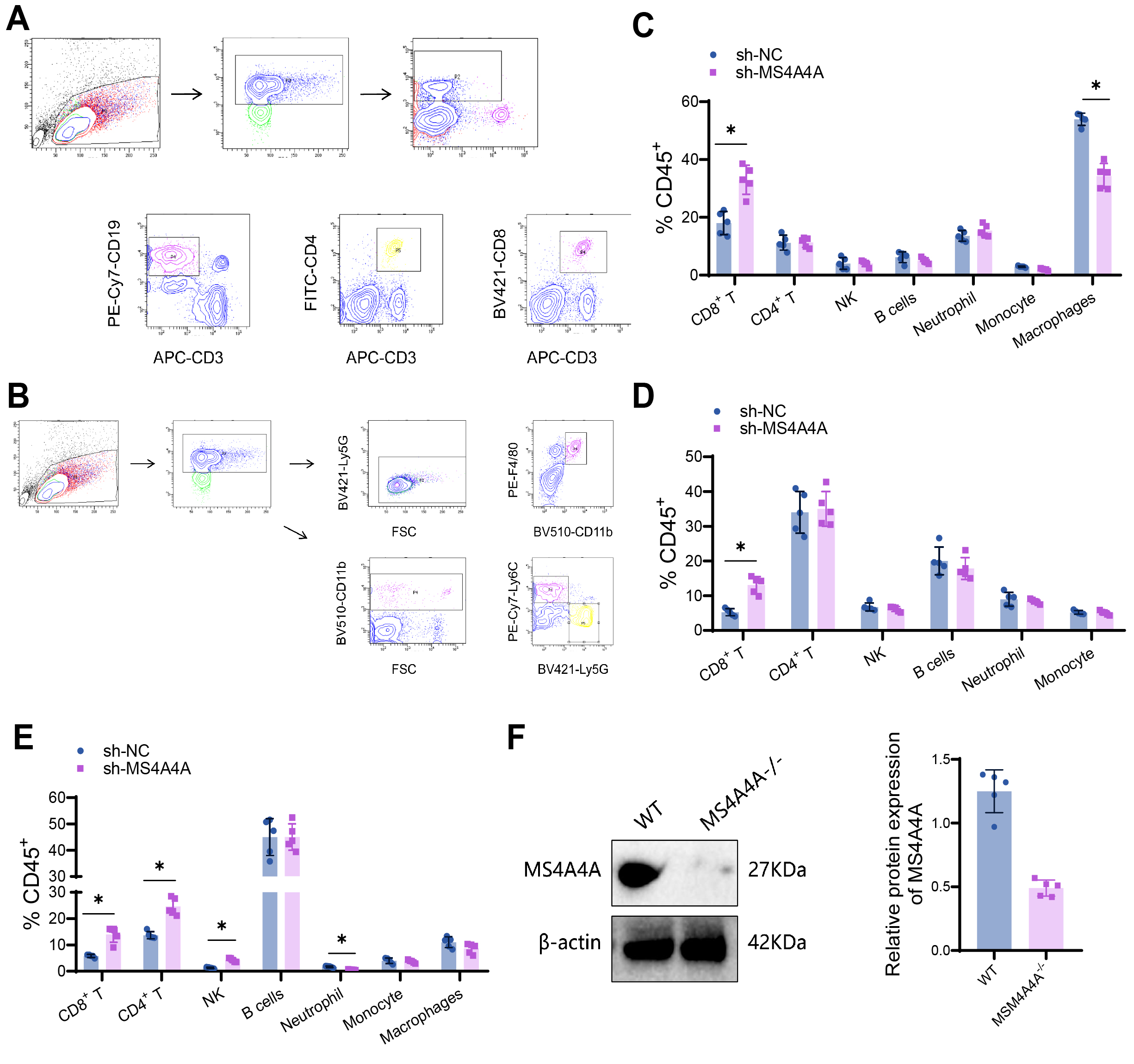

Supplement: Supplementary file 8 — Figure S8. [file CNS-30-e14791-s003.jpg]

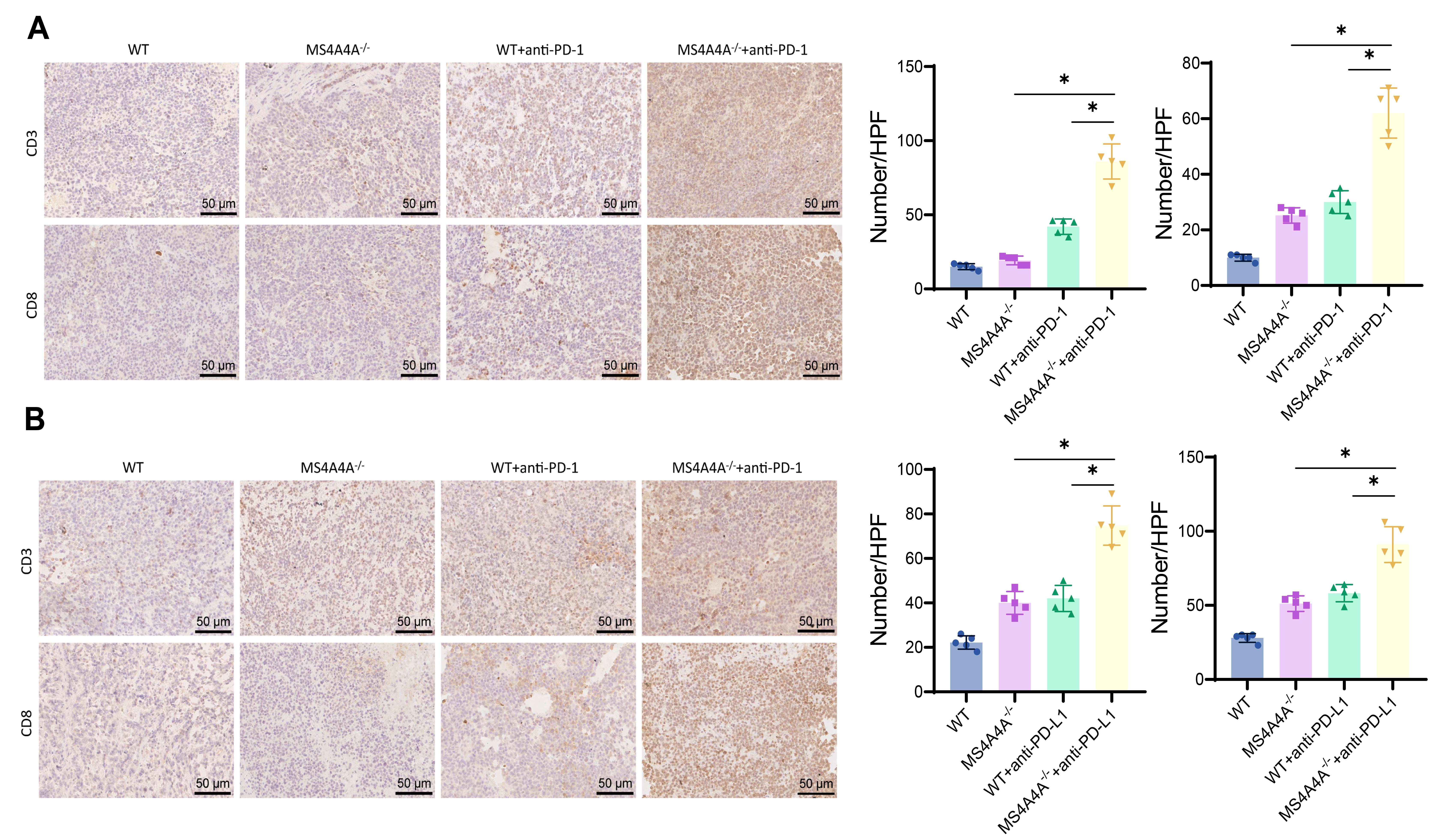

Supplement: Supplementary file 9 — Figure S9. [file CNS-30-e14791-s004.jpg]
