# Supplementary material for: Targeting MS4A4A: A novel pathway to improve immunotherapy responses in glioblastoma
Source: CNS Neurosci Ther. 2024 Jul 12;30(7):e14791. doi: 10.1111/cns.14791 (PMC11245405; doi:10.1111/cns.14791)
Supplement: Supplementary file 10 — Tables S1–S4. [file CNS-30-e14791-s006.docx]

**Table S1. shRNA interference sequence**

| Name | sh-RNA Sequences(5’-3’) |
| --- | --- |
| sh-MS4A4A-1 | GCAGAAATTGTGTTCTTAAAT |
| sh- MS4A4A-2 | ATTCTGTGAGACTTCATATTT |
| Sh-NC | GGGUGAACUCACGUCAGAA |

**Table S2. RT-qPCR primer sequences**

| **Gene** | **Primer** |
| --- | --- |
| MS4A4A  (Human) | F 5'- TGGCTGTCATACATTCACATCT -3' |
|  | R 5'- -CATTACTGACCCCCAAATTGTG -3' |
| CD163 | F 5'- TTTGTCAACTTGAGTCCCTTCAC -3' |
| (Human) | R 5'- TCCCGCTACACTTGTTTTCAC -3' |
| ARG1 | F 5'- TGGACAGACTAGGAATTGGCA -3' |
| (Human) | R 5'- CCAGTCCGTCAACATCAAAACT -3' |
| TGFB1 | F 5'- CTGTACATTGACTTCCGCAAG -3' |
| (Human) | R 5'- TGTCCAGGCTCCAAATGTAG -3' |
| β-actin | F 5'- GGACTTCGAGCAAGAGATGG -3' |
| (Human) | R 5'- AGCACTGTGTTGGCGTACAG -3' |
| MS4A4A | F 5'- ATTGGAAGCTGGCTATGGGG -3' |
| (Mouse) | R 5'- TGAGCTGATTACGATCCCCG -3 |
| MgI2 | F 5'- TTCAAGAATTGGAGGCCACT -3 |
| (Mouse) | R 5'- CAGACATCGTCATTCCAACG -3 |
| CD86 | F 5'- TAGTGAGATGGCCTGGGGAA -3 |
| (Human) | R 5'- ATCCCACCTTAGAGCCAGGT -3 |
| Tgfb1 | F 5'- AGCTGCTTATCCCAGATTCAGCCA -3 |
| (Mouse) | R 5'- TATCGAGGCCAGCTTGTTTGAGGA -3 |
| Arg1 | F 5'- CCACAGTCTGGCAGTTGGAAG -3 |
| (Mouse) | R 5'- GGTTGTCAGGGGAGTGTTGATG -3 |
| PD-1 | F 5'- CAGCTTGTCCAACTGGTCG -3 |
| (Mouse) | R 5'- GCTCAAACCATTACAGAAGGCG -3 |
| TIM3 | F 5'- TCAGGTCTTACCCTCAACTGTG -3 |
| (Mouse) | R 5'- GGCATTCTTACCAACCTCAAACA -3 |
| LAG3 | F 5'- CTGGGACTGCTTTGGGAAG -3 |
| (Mouse) | R 5'- GGTTGATGTTGCCAGATAACCC -3 |
| iNOS | F 5'- CAACAGGGAGAAAGCGCAAA -3 |
| (Mouse) | R 5'- TGATGGACCCCAAGCAAGAC -3 |
| β-actin | F 5'- AGCCATGTACGTAGCCATCC -3 |
| (Mouse) | R 5'- CTCTCAGCTGTGGTGGTGAA -3 |

Note: F, forward; R, reverse.

**Table S3. Antibody product details**

| **Name** | **Cat.** | **Dilution ratio** | **Manufacturer** | | **Country** |
| --- | --- | --- | --- | --- | --- |
| MS4A4A  (Mouse) | A18334 | 1:1000 | | ABcIonaI | CH |
| MS4A4A  (Human) | ab67134 | 1:2500 | | Abcam | USA |
| Nrf2  (Mouse) | PA5-27882 | 1:1000 | | Thermo Fisher | USA |
| SLC71AA  (Mouse) | ab314223 | 1:1000 | | Abcam | USA |
| CD206  (Mouse) | ab64693 | 1:5000 | | Abcam | USA |
| GPX4  (Mouse) | ab125066 | 1:1000 | | Abcam | USA |
| F4/80  (Mouse) | ab300421 | 1:5000 | | Abcam | USA |
| CD3  (Mouse) | ab237721 | 1:2000 | | Abcam | USA |
| CD8  (Mouse) | ab217344 | 1:2000 | | Abcam | USA |
| iNOS  (Mouse) | ab115819 | 1:2000 | | Abcam | USA |
| β-actin  (Mouse) | ab8226 | 1:1000 | | Abcam | USA |

Note: ABcIonaI (https://abclonal.com.cn/); Abcam (https://www.abcam.cn/); Thermo Fisher (https://www.thermofisher.cn/).

**Table S4. Flow cytometry labeled antibodies**

| **AntibOdies** | **SOURCE** | **IDENTIFIER** |
| --- | --- | --- |
| PE anti-mouse CD45 antibody | BioIegend | CIone no. 30-F11 |
| APC anti-mouse CD3ε antibody | BioIegend | CIone no. 145-2C11 |
| FITC anti-mouse CD8a antibody | BioIegend | CIone no. 53-6.7 |
| PE/Cyanine7 anti-mouse CD279(PD-1) antibody | BioIegend | CIone no. 29F.1A12 |
| BriIIiant vioIet 421 anti-mouse CD366 (Tim-3) antibody | BioIegend | CIone no. B8.2C12 |
| BriIIiant vioIet 421 anti-mouse Ki-67 antibody | BioIegend | CIone no.16A8 |
| BriIIiant vioIet 421 anti-mouse IFN-y antibody | BioIegend | CIone no. XMG1.2 |
| PE anti-mouse F4/80 antibody | BioIegend | CIone no. BM8 |
| APC anti-mouse CD206 antibody | Thermo Fisher Scientific | CIone no. MR6F3 |
| APC anti-mouse CD3 antibody | Thermo Fisher Scientific | CIone no. 145-2C11 |
| PerCP/Cyanine5.5 anti-mouse NK1.1antibody | BioIegend | CIone no. PK136 |
| FITC anti-mouse CD4 antibody | BioIegend | CIone no. RM4-5 |
| BriIIiant vioIet 421 anti-mouse CD8 antibody | BioIegend | CIone no. 53-6.7 |
| BriIIiant vioIet 421 anti-mouse Ly6G antibody | BioIegend | CIone no. 1A8 |
| PE-Cyanine 7 anti-mouse Ly6C antibody | BioIegend | CIone no. HK1.4 |
| PE/Cyanine7 anti-mouse CD19 antibody BioIegend | BioIegend | CIone no. 6D5 |
| BriIIiant vioIet 510 anti-mouse CD11b antibody | BioIegend | CIone no. M1/70 |
| Anti-mouse CD3ε Antibody | BioIegend | CIone no. 145-2C11 |
| InvivoMAb anti-mouse CD28 | Bio X CeII | Cat no. BE0015 |
| FITC anti-mouse CD80 Antibody | BioIegend | CIone no. 16-10A1 |
| FITC anti-mouse CD206 Antibody | BioIegend | Cione no. C068C2 |

Note: BioIegend (https://www.biolegend.com/); Thermo Fisher (https://www.thermofisher.cn/); Bio X CeII (http://www.neobioscience.com/).

**FIGURE S1 Quality control, filtering, and principal component analysis of scRNA-seq data.**

Note: (A) Quality control of each cell in the scRNA-seq data, with three scatter plots showing the number of nFeature_RNA, nCount_RNA, and percent.mt in each cell; (B) Scatter plots showing the correlation between filtered data nCount_RNA and percent.mt, and between nCount_RNA and nFeature_RNA; (C) Variance analysis to select highly variable genes in the samples (red dots represent highly variable genes, black dots represent invariant genes); (D) Principal component analysis (PCA) results for cells from different sample sources (each point represents a cell, different colors represent different samples); (E) Heatmap showing the expression levels of feature genes in the top 4 PCs of the PCA analysis, with yellow indicating upregulation and purple indicating downregulation; (F) Distribution of standard deviation of PCs, with important PCs having larger standard deviations; (G) TSNE clustering dendrogram of scRNA-seq data. n=12.

**FIGURE S2 Clustering analysis of scRNA-seq data.**

Note: (A) TSNE clustering analysis of cells grouped into 21 clusters; (B-C) Distribution of cell clusters in different samples; (D) TSNE clustering analysis of macrophages grouped into 7 clusters; (E) Distribution of samples in macrophage clusters; (F) Heatmap showing marker genes from different cell clusters; (G) Marker genes representing macrophages.

**FIGURE S3 Pseudo-time analysis of macrophages in GBM.**

Note: (A) Trajectory distribution of macrophage cell clusters in different samples over time, with the starting point on the right and the endpoint on the left; (B) Pseudo-time ordering of macrophages in a two-dimensional state space defined by Monocle 2; (C) Trajectories of three representative genes (MS4A4A, CD163, CD86).

**FIGURE S4 Quality control and PCA dimension reduction of ST data.**

Note: (A) Violin plots showing the distribution of gene counts (nFeature_Spatial), mRNA molecules (nCount_Spatial), and mitochondrial gene percentage (percent.mt) for each cell in scRNA-seq data (n=4); (B) Scatter plots showing the correlation between nCount_Spatial and percent.mt, nCount_Spatial and nFeature_Spatial, and nCount_Spatial and percent.HB in ST data (n=4); (C) Distribution of nCount_Spatial in different tissue sections of ST data (n=4), with higher expression levels represented by darker red colors; (D) Cell cycle states of each cell in ST data, where S.Score represents S phase and G2M.The score represents G2M phase (n=4); (E) SCTransform and LogNormalize normalized results in ST data (n=4); (F) Heatmap showing the top 20 highly correlated genes in PCA for PC_1 – PC_6, with yellow indicating upregulation and purple indicating downregulation (n=4).

**FIGURE S5 Inhibition of MS4A4A in TAMs slows down GBM progression *in vitro*.**

Note: (A) RT-qPCR and western blot analysis of MS4A4A knockdown; (B) CCK8 assay measuring the proliferation capacity of different groups of GBM cancer cells with TAM supernatant; (C) Edu assay measuring the proliferation activity of different groups of cancer cells with TAM supernatant; (D) Transwell assay measuring the invasion of different groups of cancer cells with TAM supernatant; (E-F) Scratch assay measuring the migration of different groups of cancer cells with TAM supernatant; * indicates p < 0.05 when comparing the two groups, all cell experiments were repeated three times.

**FIGURE S6 Inhibition of MS4A4A suppresses the infiltration of M2 macrophages and inhibits GBM progression by activating the TAM-mediated ferroptosis pathway.**

Note: (A-C) Investigation of the effect of MS4A4A expression on macrophage polarization. (A) Bone marrow cells were extracted from C57BL/6N mice and induced to generate bone marrow-derived macrophages (BMDMs) using L929 cell-conditioned medium (L929-CM). On the 6th day, BMDMs were transfected with MS4A4A knockdown plasmid or control plasmid by lentivirus and then cultured in CT2A cell-conditioned medium (CT2A-CM) or GL261 cell-conditioned medium (GL261-CM) for 24 hours. (B-C) qRT-PCR was used to measure the expression levels of MS4A4A and ARG1 (n=3); (D-E) Flow cytometry analysis of the proportion of M2 macrophages in different groups of macrophages cultured in CT2A cell-conditioned medium (n=3); (F-G) Flow cytometry analysis of the proportion of M2 macrophages in different groups of macrophages cultured in GL261 cell-conditioned medium (n=3); (H) RT-qPCR and western blot analysis of MS4A4A overexpression in macrophages cultured in CT2A cell-conditioned medium (n=3); (I) Western blot analysis of MS4A4A overexpression in macrophages cultured in GL261 cell-conditioned medium (n=3); (J) Expression levels of anti-inflammatory cytokines IL-10 and TGF-β1 in CT2A TAM supernatant from MS4A4A knockdown group (n=3); (K) Expression levels of anti-inflammatory cytokines IL-10 and TGF-β1 in GL261 TAM supernatant from MS4A4A knockdown group (n=3); * indicates p < 0.05, all cell experiments were repeated three times.

**FIGURE S7 Inhibition of macrophage MS4A4A delays GBM progression *in vivo*.**

Note: (A-B) Tumor growth after intracranial/subcutaneous injection (s.c.) of GL261 cells in WT and MS4A4A-/- mice (n=5/group); (C-F) FACS analysis of infiltrating CD8+ T cells and TAM-specific marker expression in GL261-tumor-bearing mice; (G) Immunohistochemical staining using CD206 and iNOS antibodies to detect infiltration of CD206+ and iNOS+ macrophages in GL261 subcutaneous tumors. The number of CD206+ and iNOS+ cells per high-power field (HPF) in each group of mouse subcutaneous tumor sections was counted, and 5 HPFs were randomly selected for analysis on each slide; (H) qRT-PCR detection of relative gene expression levels; (I) WT and MS4A4A-/- mice were injected subcutaneously with GL261 cells on day 0, and then received intraperitoneal injection of PBS liposomes (PL) or clodronate liposomes (CL) to deplete macrophages on the previous day and days 3, 7, 10, 16, and 17 after tumor cell implantation (n=5); (J) Immunohistochemical staining using F4/80-specific antibody to detect the number of macrophages in the spleen and tumor of MS4A4A-/- mice with GL261 subcutaneous tumors. The number of F4/80+ cells per HPF in each group of mouse subcutaneous tumor sections was counted, and 5 HPFs were randomly selected for analysis on each slide. * indicates p < 0.05, n=5.

**FIGURE S8 Enhanced anti-tumor immunity in MS4A4A-deficient mice.**

Note: (A-E) Composition analysis of tumor, peripheral blood, and spleen samples in WT and MS4A4A-/- mice using different combinations of antibodies based on different cell surface markers (NK cells, B cells, T cells, macrophages, and monocytes); (F) Western blot analysis of MS4A4A expression in peripheral blood of MS4A4A-deficient mice and WT mice to check the knockout efficiency. * indicates p < 0.05, n=5.

**FIGURE S9 Representative images and statistical analysis of immunohistochemical staining of CD3 and CD8 in tumor sections.**

Note: (A) Representative images and statistical analysis of immunohistochemical staining of CD3 and CD8 in subcutaneous tumor sections of CT2A tumor-bearing mice after combined MS4A4A knockout and PD-1 treatment; (B) Representative images and statistical analysis of immunohistochemical staining of CD3 and CD8 in subcutaneous tumor sections of CT2A tumor-bearing mice after combined MS4A4A knockout and PD-L1 treatment. * indicates p < 0.05 when comparing between the two groups, n=5.
